# Hidden diversity in the *Trichostomum brachydontium* complex (Pottiaceae, Bryophyta) revealed by integrative taxonomy

**DOI:** 10.3389/fpls.2026.1822444

**Published:** 2026-04-21

**Authors:** Omar Rodríguez, M. Teresa Gallego, Juan A. Jiménez, María J. Cano

**Affiliations:** Departamento de Biología Vegetal (Botánica), Facultad de Biología, Universidad de Murcia, Murcia, Spain

**Keywords:** bryophytes, integrative taxonomy, Pottiaceae, South America, *Trichostomum*

## Abstract

We applied an integrative taxonomic approach combining multilocus phylogenetics, morphoanatomical analyses, and multivariate statistics to reassess the diversity of the *Trichostomum brachydontium* complex in South America. Analyses of five molecular markers (ITS, *atpB–rbcL*, *trnG*, *trnL–F*, *rps4*) and 67 morphological characters revealed that specimens traditionally identified as *T. brachydontium* represent 11 distinct lineages, none matching the diagnostic traits of *T. brachydontium* sensu stricto. Accordingly, *T. brachydontium* is excluded from the South American flora. Ten lineages exhibit significant phenotypic differentiation supported by PCA, LDA (accuracy = 96.6%), and hierarchical clustering. Until further studies, four of them are recognized as *T. antillarum*, *T. basilatinervium*, *T. hondurense* and *T. termitarum*. Our results highlight cryptic speciation and biogeographic divergence between European and South American populations, underscoring the need for taxonomic revision. Additionally, phylogenetic analyses confirm the placement of *T. involutum* within the genus *Trichostomum*. We propose a new combination: *Neotrichostomum jamaicense* (Mitt.) M.J. Cano, M.T. Gallego & Omar Rodr., comb. nov. The known distribution of *Trichostomum antillarum* is here substantially expanded across South America, *T. hondurense* is newly recorded in South America, and *T. termitarum* is reported from Argentina for the first time.

## 1 Introduction

Advances in phylogenetic methods and molecular data have significantly improved our understanding of evolutionary relationships in plants. However, an exclusive reliance on molecular evidence often overshadows morphological and anatomical analyses, especially in taxa with few diagnostic traits (Vanderpoorten and Shaw, 2010; Hedenäs, 2011; Hirano et al., 2014). Rather than competing, these approaches are complementary (Dayrat, 2005). Integrative taxonomy, which combines molecular, morphological, and ecological data, among others, provides a robust framework for species delimitation and is especially valuable for groups rich in cryptic species, such as bryophytes (Patel et al., 2021).

The moss family Pottiaceae Hampe exemplifies this complexity. It is one of the most diverse families of mosses, comprising over 1200 species worldwide (Brinda and Atwood, 2026), with peak diversity in tropical America (Zander, 1993). Molecular techniques have clarified relationships within several genera (e.g., Werner et al., 2002; Alonso et al., 2016; Cano et al., 2022; Gallego et al., 2022; Jiménez et al., 2022), but many remain poorly understood. Within Pottiaceae, the subfamily Trichostomoideae Broth. stands out for its taxonomic challenges. Phylogenetic evidence supports its monophyly (Werner et al., 2005) and division into three tribes, Hyophileae M. Fleisch., Pleuroweisieae Limpr., and Trichostomeae Mont. ex Berk (Köckinger and Kučera, 2011), although the limits of certain taxa remain unresolved and many genera are still insufficiently studied taxonomically.

The genus *Trichostomum* Bruch comprises a diverse group of mosses belonging to the subfamily Trichostomoideae. It was first described by Philipp Bruch in 1829 (Müller, 1829; Dixon, 1939). Currently, 81 species are accepted worldwide (Brinda and Atwood, 2026), although nearly 700 names have been published for the genus (Tropicos.org, 2026), indicating a long history of taxonomic instability. Approximately one third of the recognized species occur in tropical America (Gradstein et al., 2001), yet most South American taxa remain poorly known and are represented only by type specimens, with few or no additional studies (Crosby et al., 1999). **Table 1** summarizes the 25 *Trichostomum* species currently accepted for South America and their distribution (Brinda and Atwood, 2026), including several taxa known exclusively from type material and others that remain poorly understood, such as *Trichostomum compactulum* Müll. Hal (Crosby et al., 1999).

**Table 1:** Distribution of currently accepted *Trichostomum* species cited in South America.

Taxa	Distribution in South America
*Trichostomum aequatoriale Spruce ex Dixon	Ecuador (Dixon, 1924)
*Trichostomum antillarum* R.H. Zander	Brazil (Zander, 2023)
*Trichostomum apophysatulum Herzog	Bolivia (Herzog, 1916)
*Trichostomum bellii E.B. Bartram	Ecuador (Bartram, 1955)
*Trichostomum basilatinervium* M.J.Cano, M.T.Gallego, Omar Rodr. & Larraín	Chile (Rodríguez et al., 2026)
*Trichostomum brachydontium* Bruch	Argentina (Hübschmann, 1986), Bolivia (Churchill et al., 2009), Brazil (Pôrto, 1990), Chile (Brotherus, 1924), Colombia (Robinson, 1967), Ecuador (Mitten, 1869), Peru (William, 1916), Venezuela (Griffin, 1977)
*Trichostomum compactulum* Müll. Hal.	Argentina (Müller, 1879), Bolivia (Herzog, 1910)
*Trichostomum edentulum Broth.	Bolivia (Herzog, 1916)
*Trichostomum elliottii Broth. ex Dusén	Chile (Dusén, 1906)
*Trichostomum gracillimum Müll. Hal.	Argentina (Müller, 1879)
*Trichostomum involutum* Sull.	Ecuador (Gradstein and Weber, 1982), Bolivia (Hermann, 1976)
*Trichostomum lambii E.B. Bartram	Argentina (Bartram, 1965)
*Trichostomum lindigii (Hampe) R.H. Zander (=*Systegium lindigii* Hampe)	Colombia (Hampe, 1865)
*Trichostomum loxorhynchum* (Müll. Hal. ex Ångstr.) M.J. Cano, M.T. Gallego & Omar Rodr. (=*Gymnostomum jamesonii* Arn.)	Bolivia (Rodríguez et al., 2025), Brazil (Arnott, 1824), Paraguay (Rodríguez et al., 2025)
*Trichostomum mammillosum* R.H. Zander	Brazil (Ellis et al., 2025)
*Trichostomum mittenianum R.H. Zander (=*Weissia umbrosa* Mitt.)	Ecuador, Peru (Mitten, 1869)
*Trichostomum ovatifolium R.H. Zander (=*Hymenostomum anomalum* Broth.)	Bolivia (Herzog, 1916)
*Trichostomum paludicola (Broth.) Hilp.	Bolivia (Herzog, 1916)
*Trichostomum plicatulum Müll. Hal.	Argentina (Müller, 1882)
*Trichostomum pomangium Herzog	Bolivia (Herzog, 1916)
*Trichostomum termitarum* (Müll. Hal.) R.H. Zander	Brazil (Müller, 1900)
*Trichostomum tovarense Müll. Hal.	Venezuela (Müller, 1897)
*Trichostomum tucumanense E.B. Bartram	Argentina (Bartram, 1965)
*Trichostomum urceolare (Hampe) R.H. Zander	Brazil (Hampe, 1870)
*Trichostomum williamsii R.H. Zander (=*Astomum chilense* R.S. Williams)	Chile (William, 1915)

Species known only from original material are indicated with an asterisk. Only the reference of the first record per country is cited.

Morphologically, *Trichostomum* is characterized by leaves with flat or incurved margins, a costa with two distinct stereid bands, a yellow to orange-yellow KOH reaction, and stems with a well-developed central strand (Zander, 1993; Guerra, 2006). These characters have traditionally played a central role in species delimitation within the genus.

The classification of *Trichostomum* has undergone numerous revisions since its original description (Mitten, 1869; Lindberg, 1879; Kindberg, 1898; Andrews, 1945). Over time, several species originally placed in *Trichostomum* have been transferred to other genera within Pottiaceae based on morphological or molecular evidence (e.g., Jiménez, 2006; Cano and Gallego, 2008; Cano and Jiménez, 2013; Alonso et al., 2018; Müller et al., 2018), while other taxa have been incorporated into *Trichostomum* (Thouvenot et al., 2018; Thouvenot and Müller, 2021). Despite this extensive taxonomic flux, no comprehensive global revision of the genus exists, and current knowledge is largely derived from regional studies conducted in Japan (Saito, 1975), southern Africa (Sim, 1926; Magill, 1981), India (Aziz and Vohra, 2008), China (Li and Iwatsuki, 1996), the Iberian Peninsula (Guerra, 2006), Mesoamerica (Allen, 2002), and South America (Churchill and Linares, 1995; Churchill et al., 2009; Cano et al., 2020).

Within this context, *Trichostomum brachydontium* Bruch stands out as one of the most widespread and morphologically variable species in the genus. It occurs on all continents except Antarctica (Zander, 2007) and is characterized by stems with sclerodermis and a well-developed central strand, ligulate to lanceolate leaves with flat margins, an excurrent costa ending in a mucro, two distinct stereid bands, and a peristome with short, straight or rudimentary teeth (Zander, 1993; Allen, 2002; Guerra, 2006; Ros et al., 2022). The species has 88 synonyms (WFO, 2026) and 22 recognized infraspecific taxa (Tropicos.org, 2026), although most bryological floras do not currently recognize infraspecific ranks for this taxon (Saito, 1975; Li and Iwatsuki, 1996; Allen, 2002; Smith, 2004; Guerra, 2006). Its extreme morphological variability and broad distribution have contributed to longstanding taxonomic difficulties and to the recognition of the *T. brachydontium* complex as one of the most problematic groups within *Trichostomum*.

Ros et al. (2022) showed that the pronounced morphological variability in European populations of *T. brachydontium* corresponds to genetic diversity. This integrative approach vindicates Herzog’s early work (Herzog, 1907), as several infraspecific taxa he characterized have now been reinstated at species rank (*Trichostomum littorale* Mitt., *T. meridionale* Ros, O. Werner and R.D. Porley, and *T. herzogii* Ros, O. Werner and R.D. Porley) or retained as a variety level (*T. brachydontium* var. *cylindricum* (Schimp.) Cout.). Except for this phylogenetic study, the *T. brachydontium* species complex has not been directly addressed in phylogenetic research and appears only as an outgroup in analyses of other Trichostomoideae genera (e.g., Werner et al., 2005; Grundmann et al., 2006; Alonso et al., 2016). Its variability in the Southern Hemisphere remains undocumented, and no comprehensive phylogenetic hypothesis exists for Trichostomoideae. Available studies provide only partial characterizations of select genera, limiting broader evolutionary insights within Pottiaceae. In this context, our objectives were to: (1) analyze morphological variability of the *T. brachydontium* complex using multivariate statistics; (2) assess phylogenetic relationships among Trichostomoideae genera using nuclear and chloroplast markers; (3) determine the phylogenetic position of *Trichostomum* within the subfamily; and (4) resolve relationships among potential cryptic or pseudo-cryptic species of *T. brachydontium* in South America using molecular data.

## 2 Materials and methods

### 2.1 Taxon sampling

A total of 108 specimens were selected to capture the morphological variability and geographical range of the *T. brachydontium* complex. Most were collected during fieldwork by our research group in Argentina, Bolivia, Brazil, Ecuador, Peru, Venezuela, and Chile between 2001 and 2017. Complete locality details are available on the group’s website (www.pottiaceae.com). Voucher specimens are deposited in MUB, CORD, LPB, UB, LOJA, USM, VEN and CONC herbaria. Additional material from international herbaria, including available type specimens, were examined (B, BM, FH, JE, KRAM, L, M, MO, NY and S). The dataset also included seven *T. brachydontium* specimens from outside South America (Spain, Italy, Azores), and 19 specimens representing other species of *Trichostomum*: *T. antillarum* R.H. Zander (2), *T. herzogii* (4), *T. hondurense* B.H. Allen (1), *T. involutum* Sull. (2), *T. littorale* (4), *T. meridionale* (4), and *T. platyphyllum* (Broth. ex Ihsiba) P.C. Chen (2). Four specimens of the recently described *Trichostomum basilatinervium* M.J.Cano, M.T.Gallego, Omar Rodr. & Larraín (Rodríguez et al., 2026) were also included, as many had previously been misidentified as *T. brachydontium*. Their inclusion allowed evaluation of phylogenetic relationships and discrimination patterns in morphological analyses, aiding the identification of additional potential species. The recently described species *T. mammillosum* R.H. Zander (Zander, 2023) was excluded from this study because sequencing was not possible and its morphology differs from that of *T. brachydontium* complex.

To establish the phylogenetic position of *Trichostomum* within Trichostomoideae, 25 genera representing the three tribes were sampled: Hyophileae (*Hyophila* Brid., *Plaubelia* Brid.), Pleuroweisieae (*Anoectangium* Schwägr., *Gymnostomum* Hedw., *Gyroweisia* Schimp., *Hymenostyliella* E.B. Bartram, *Hymenostylium* Lindb., *Leptobarbula* Schimp., *Molendoa* Lindb., *Reimersia* P.C. Chen, *Tuerckheimia* Broth.) and Trichostomeae (*Anaschisma* R.H. Zander, *Aschisma* Lindb., *Chionoloma* Dixon, *Ephemerum* Hampe, *Eucladium* Bruch and Schimp., *Neotrichostomum* R.H. Zander, *Pachyneuropsis* H. Mill., *Pleurochaete* Lindb., *Pottiopsis* Blockeel, *Streptocalypta* Müll. Hal., and A.J.E. Sm., *Tortella* (Müll. Hal.) Limpr., *Weissia* Hedw.). Additionally, two specimens of *Tainoa* R.H. Zander and four of *Hydrogonium* (Müll. Hal.) A. Jaeger were included, as molecular data for these genera are scarce and their placement remains uncertain. The selection of genera followed previous phylogenetics studies of Trichostomoideae (e.g., Werner et al., 2004, 2005; Alonso et al., 2016; Inoue and Aung, 2021). *Scopelophila cataractae* (Mitt.) Broth. served as the outgroup.

Specimen data, including herbarium information and GenBank accession numbers, are provided in **Supplementary Table 1**. Newly generated sequences (482 in total) are indicated in bold. Species nomenclature follows Brinda and Atwood (2026), except for *Neotrichostomum crispulum* (Bruch) R.H. Zander and *Scopelophila cataractae*, which follow Zander (1993, 2023), respectively.

### 2.2 DNA extraction, amplification, and sequencing

Plants were placed under a stereo microscope and the green distal portion of a few gametophores per specimen were dissected. Total genomic DNA was extracted using the method of Suzuki et al. (2013) with some minor modification (see Rodríguez et al., 2025).

Five loci were selected: four chloroplast regions, *atp*B-*rbc*L intergenic spacer, region (*atp*B-*rbc*L), *trn*GUCC G2 intron (*trn*G), *trn*LUAA exon *trn*FGAA region (*trn*L-F), and gene *rps*4-*trn*S (*rps*4), and the nuclear internal transcribed spacers 1 and 2 (ITS1-5.8S-ITS2). These loci have proven effective for phylogenetic reconstruction in Pottiaceae (e.g., Cano et al., 2009; Alonso et al., 2016; Cano et al., 2022; Gallego et al., 2022; Jiménez et al., 2022; Cano et al., 2024). PCR amplification and sequencing protocols followed Cano et al. (2024) for *atp*B-*rbc*L, *trn*G, *trn*L-F, and ITS1-5.8S-ITS2, and Cano et al. (2022) for *rps*4.

### 2.3 Phylogenetic analysis

Phylogenetic relationships were inferred following Rodríguez et al. (2025). Neighbor-Net (NN) networks were constructed using SplitsTree v4.14.8 (Huson and Bryant, 2006) from a combined nuclear and chloroplast dataset, with 1000 bootstrap replicates. Nodes with support ≥80% were considered strongly supported. This approach allowed clustering of individuals by genetic distance and provided a visual representation of relationships and affinities.

### 2.4 Morphological-anatomical analysis

After establishing molecular relationships within *T. brachydontium* s.l., a detailed morphological study was performed on specimens corresponding to the identified lineages.

Morphological and anatomical examinations followed standard protocols for Pottiaceae (Zander, 1993). Microscopic examinations and measurements were conducted using an Olympus BH-2 light microscope, and microphotographs were captured with a Jenoptik ProgRes C7 camera mounted on the microscope. Measurements of laminal cells and characterization of basal laminal cell types followed Alonso et al. (2018), although the ‘transition zone’ character is defined as the percentage of the leaf occupied by transition cells, i.e. the cells between the papillose laminal cells and the smooth basal laminal cells. Specimens were examined in 2% potassium hydroxide (Zander, 1993).

Three plants per collection were dissected for measurements. A total of 67 gametophyte and 31 sporophyte characters were evaluated per plant. Unfortunately, most specimens lacked mature sporophytes, preventing complete data collection and leading to their exclusion from the statistical analyses.

### 2.5 Statistical analysis

Statistical analyses were performed on the morphometric dataset comprising the 108 intragroup specimens included in the molecular phylogeny (**Supplementary Table 1**). Specimens were classified into 11 groups based on the results of the molecular phylogenetic analysis, according to their placement within well-supported clades (UFBoot ≥ 95%; aBayes ≥ 0.95) recovered in the inferred phylogenetic tree.

A total of 41 quantitative and 26 qualitative variables were used to characterize each specimen, following standardized protocols for Pottiaceae. Variables were coded numerically (1–67; see **Supplementary Table 2**) and standardized (mean = 0, standard deviation = 1). Qualitative variables were treated as nominal and coded into states (**Supplementary Tables 2**, **3**). Analyses were performed in R v.4.4.3 (R Core Team, 2025).

Multivariate analyses included Principal component analysis (PCA) on quantitative data to visualize morphological variation, and Linear discriminant analysis (LDA) on combined dataset to assess trait discrimination among the 11 groups defined *a priori* based on well-supported clades in the molecular phylogeny. LDA was validated using 10-fold cross-validation; accuracy ≥90% and Kappa ≥0.9 indicated strong agreement (Cohen, 1960; Landis and Koch, 1977; Kuhn and Johnson, 2013). Results were interpreted using the first three discriminant functions. PCA and LDA were visualized with scatterplots and 90% confidence ellipses. Additionally, hierarchical cluster analysis (HCA) using Ward’s method and a Euclidean distance tested congruence between morphology-based clusters and the 11 genetic groups identified in the molecular phylogeny. Box plots were generated for the most discriminating quantitative variables to illustrate variability across clades.

## 3 Results

### 3.1 Molecular analyses

**Figure 1:**
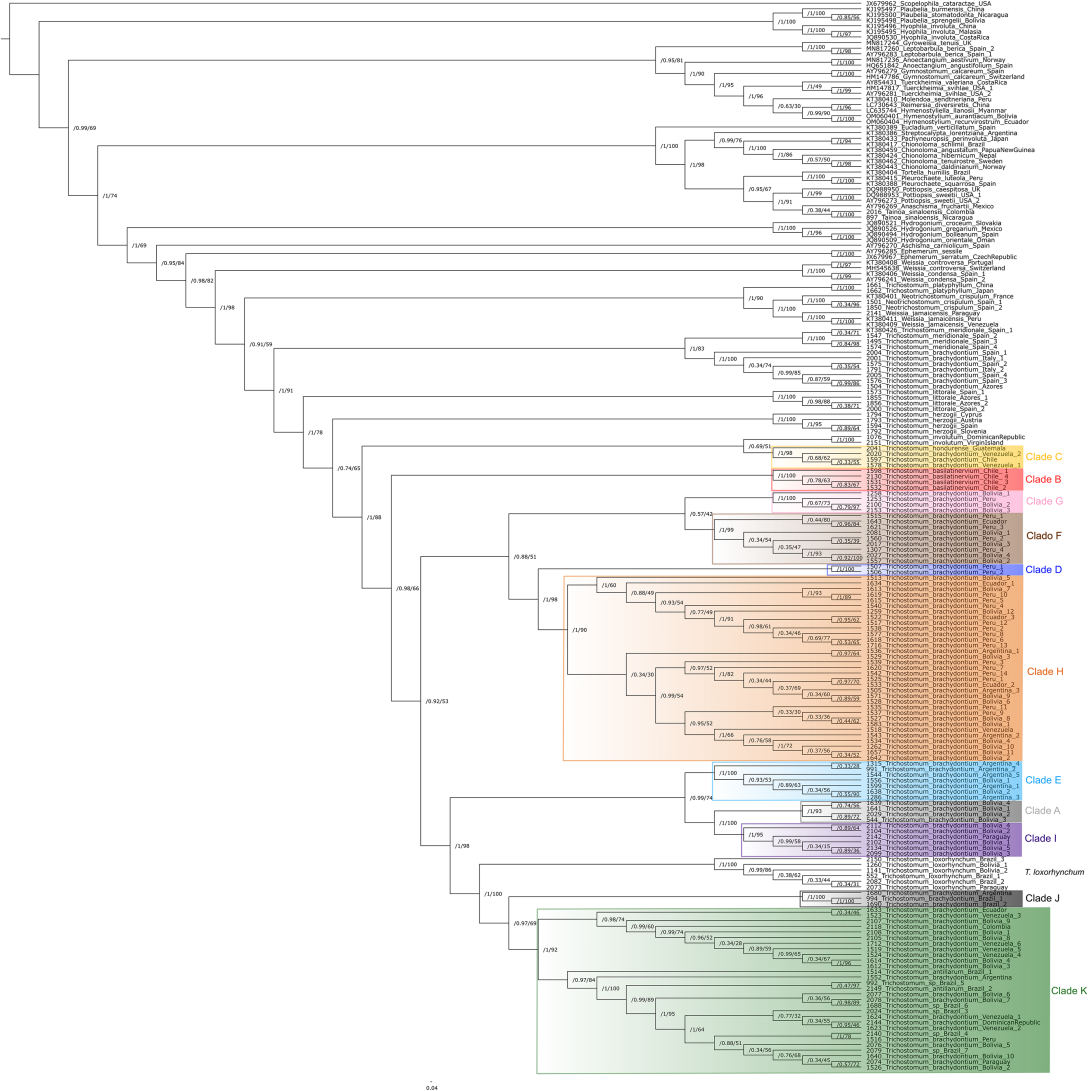
Maximum likelihood (ML) tree inferred from the concatenated nuclear and plastid dataset using IQ-TREE v.2.3.4, showing the phylogenetic relationships among all specimens included in the study. Numbers at the nodes indicate support values: aBayes (left) and UFBoot (%) (right).

Phylogenetic analyses were conducted on a combined dataset of 190 specimens, totaling 4023 aligned base pairs (5212 included indels). **Table 2** summarizes the characteristics of each dataset. Individual nuclear and chloroplast trees (**Supplementary Figures S1**, **S2**) and the concatenated tree (**Figure 1**) showed congruent topologies, with most nodes strongly supported (UFBoot ≥ 95%; aBayes ≥ 0.95), confirming the robustness of inferred relationships.

**Table 2:** Summary statistics for nuclear (DN) and chloroplast (DC) datasets analyzed in this study.

Locus	Number of specimens	Newly sequences generated	Length of the sequence	Parsimony information	Variable sites
ITS	190	138	1490	416 (33.29%)	725 (48.65%)
*atp*B**-***rbc*L	116	97	645	79 (12.25%)	153 (23.72%)
*trn*G	98	79	694	86 (12.39%)	216 (31.12%)
*trn*L-F	122	101	497	59 (11.87%)	125 (25.15%)
*rps*4	96	67	697	107 (15.35%)	221 (31.70%)
DN+DC	190	482	4023	747 (18.57%)	1442 (35.84%)

The only notable incongruence involved *T. platyphyllum*: resolved within *Trichostomum* in the nrITS tree (aBayes = 1; UFBoot = 69), but placed outside in chloroplast and concatenated trees (aBayes = 1; UFBoot = 91), as sister to *Neotrichostomum crispulum* plus *Weissia jamaicensis* (Mitt.) Grout.

Concatenated analyses revealed major clades within Pottiaceae: (1) *Hyophila* and *Plaubelia* (aBayes = 1; UFBoot = 100); (2) *Anoectangium*, *Gymnostomum*, *Gyroweisia*, *Hymenostyliella*, *Hymenostylium*, *Leptobarbula*, *Molendoa*, *Reimersia* and *Tuerckheimia* (aBayes = 0.95; UFBoot = 81); (3) *Anaschisma*, *Chionoloma*, *Eucladium*, *Pachyneuropsis*, *Pleurochaete*, *Pottiopsis*, *Streptocalypta*, *Tainoa* and *Tortella* (aBayes = 1; UFBoot = 100); (4) *Aschisma*, *Ephemerum*, *Neotrichostomum*, *Trichostomum* and *Weissia*, (aBayes = 0.95; UFBoot = 84). *Weissia* splits into two clades: *W. controversa* Hedw. and *W. condensa* (Voit) Lindb. (aBayes = 1; UFBoot = 100) and *W. jamaicensis* clustering with *Neotrichostomum crispulum* (aBayes = 1; UFBoot = 100). The genus *Trichostomum* is resolved with significant support from aBayes (aBayes = 1; UFBoot = 91), with *T. platyphyllum* recovered as sister to a clade comprising *Neotrichostomum crispulum* and *Weissia jamaicensis* (aBayes = 1; UFBoot = 90). *Trichostomum* is well supported (aBayes = 1; UFBoot = 91), but *T. platyphyllum* forms a separate clade with *Neotrichostomum crispulum* and *W. jamaicensis* (aBayes = 1; UFBoot = 90). Relationships among *Trichostomum* species are strongly supported (aBayes = 1; UFBoot = 100), including *T. meridionale*, *T. littorale*, *T. herzogii*, *T. involutum*, and *T. loxorhynchum* (Müll. Hal. ex Ångstr.) M.J. Cano, M.T. Gallego & Omar Rodr. In contrast, *T. brachydontium* exhibits marked geographic divergence: European/Macaronesian specimens cluster with other European species (aBayes = 1; UFBoot = 100), while South American specimens group with *T. involutum*, *T. loxorhynchum* and *T. antillarum* (aBayes = 1; UFBoot = 88). The South American group of *T. brachydontium* revealed a complex structure, forming 11 distinct lineages (A–K). Clade B corresponds to the recently described *T. basilatinervium* (Rodríguez et al., 2025). Most clades (D, E, G, J) and *T. basilatinervium*, have maximal support (aBayes = 1; UFBoot = 100); Clades C, F, and I show strong support (aBayes = 1; UFBoot ≥ 95); while A, H, and K are supported mainly by aBayes (aBayes = 1; UFBoot < 95). *Trichostomum hondurense* falls within Clade C (aBayes = 1; UFBoot = 98), and *T. antillarum* falls within Clade K (aBayes = 1; UFBoot = 92).

**Figure 2:**
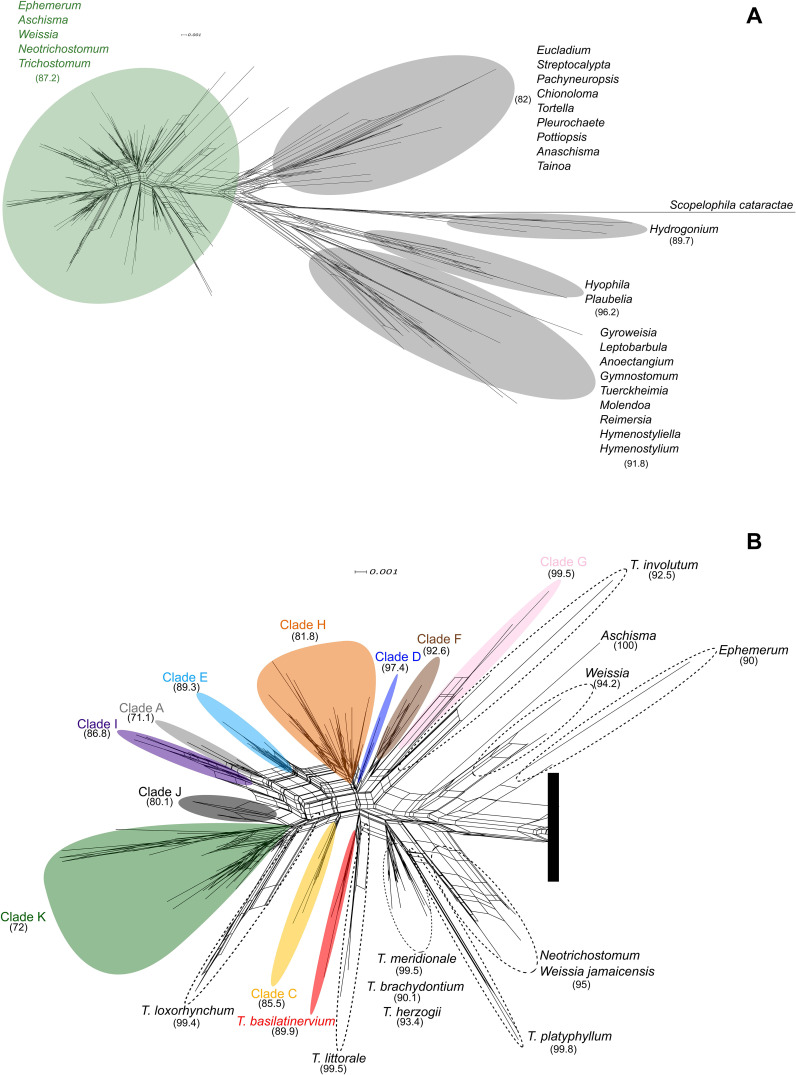
Neighbor-Net (NN) network based on the combined nuclear and plastid dataset, generated in SplitsTree from a genetic distance matrix. Support for splits was assessed by bootstrap (1000 replicates); values in parentheses indicate bootstrap percentages, with ≥80% considered well supported. **(A)** Complete network. **(B)** Detail of tribe Trichostomeae. Colors denote lineages within the *Trichostomum brachydontium* complex in South America.

SplitsTree network analyses (**Figures 2A, B**) largely agree with ML results (**Figure 1**). *Hydrogonium* is clearly positioned as an independent lineage (bootstrap = 89.7%), and *Hyophila* and *Plaubelia* form a distinct group (bootstrap = 96.2%). Two additional well-supported clusters are evident: *Anoectangium*, *Gymnostomum*, *Gyroweisia*, *Hymenostyliella*, *Hymenostylium*, *Leptobarbula*, *Molendoa*, *Reimersia* and *Tuerckheimia* (bootstrap values = 91.8%), and *Anaschisma*, *Chionoloma*, *Eucladium*, *Pachyneuropsis*, *Pleurochaete*, *Pottiopsis*, *Streptocalypta*, *Tainoa* and *Tortella* (bootstrap = 82%). Finally, *Aschisma*, *Ephemerum*, *Neotrichostomum*, *Trichostomum* and *Weissia* form a separate group (bootstrap = 87.2%).

Within the *T. brachydontium* complex, network reticulations highlight evolutionary complexity (**Figure 2B**). Clades A, E, I, J, and H exhibit extensive reticulations, suggesting complex evolutionary histories, whereas *T. basilatinervium* and Clades C, D, and F show more defined and less conflicting relationships. The close proximity of Clades A and I may indicate recent divergence or high homoplasy. Clades J and G, with limited sampling, display highly conflict, while Clade F, with more specimens, shows a linear and well-resolved pattern. Clade K contains two internally divergent groupings, potentially indicating cryptic speciation.

### 3.2 Statistical analyses

#### 3.2.1 Principal component analysis

**Figure 3:**
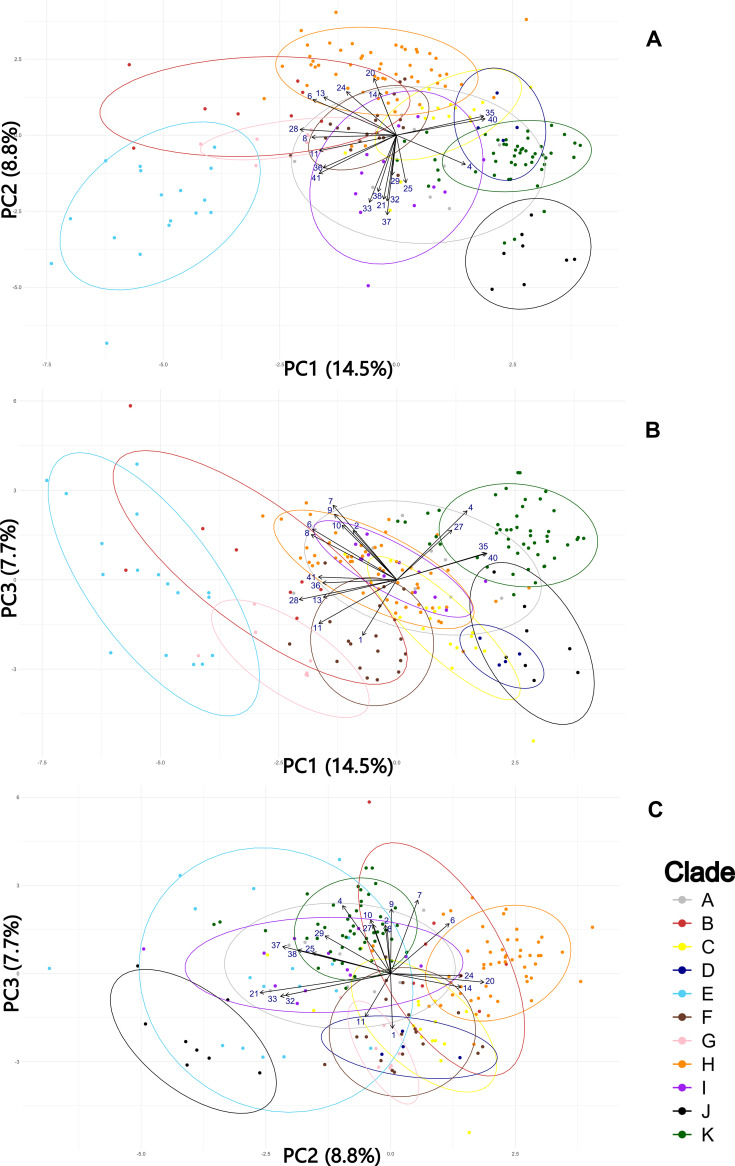
Principal Component Analysis (PCA) of South American specimens of *Trichostomum brachydontium*. Colors indicate clades identified in the molecular phylogeny (A–K). Points represent specimens. Ellipses show 90% confidence intervals around group means. **(A)** PC1 vs PC2; **(B)** PC1 vs PC3; **(C)** PC2 vs PC3.

The PCA results indicated that the first three principal components (PCs) explain 31% of the variance. Contributions of each quantitative character to the PCA axes are summarized in **Supplementary Figure S3**. For clarity, only the ten most influential variables for each of the first three PCs have been represented as vectors in the PCAs plots (**Figures 3A–C**). These figures show substantial overlap among the identified phylogenetic clades, with no clade completely isolated from the other. The most influential variables in PC1 were cell papillosity and papilla size (35, 36, 40, 41), length of the juxtacostal basal cells and mucro (28, 11), leaf width (6, 8), number of guide cells in the middle zone (13) and percentage of border extension (4). In PC2, the most influential variables were the percentage of basal hyaline and transition zone occupation (20, 21), size of the marginal cells (24, 25, 32, 33, 37, 38) and number of guide cells in the upper zone (14). For PC3, the most influential variables were leaf and costa width (6, 7, 8, 9, 10), percentage of border extension (4), plant and mucro length (1, 11), width of central basal cells (27), and stem diameter (2).

**Figure 4:**
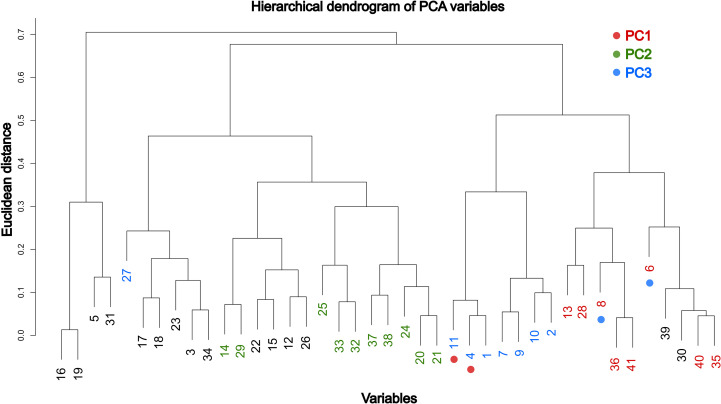
Hierarchical dendrogram based on Euclidean distances of quantitative variables with absolute loadings > 0.4 in any component. Highlighted colors on the scale identify the variables with the greatest influence in each of the first three principal components (PC1–PC3). Variables with significant importance across multiple principal components are marked with a dot and colored according to the scale.

Finally, to assess whether the original grouping of quantitative variables corresponded to the directions of maximum variance in the PCA, a hierarchical dendrogram was constructed (**Figure 4**). All significant variables for PC1 are grouped in a single clade, except variables 4 and 11, which fall within the PC3 clade. The most influential variables for PC2 are also grouped together, except for variables 14 and 29, which are separated in the dendrogram along with variables that have little effect on the first three components. Variables with the highest percentage contribution to PC3 are included in the same clade, except for variables 6 and 8 (significant for PC1) and variable 27, which is isolated with other less influential variables on the first three components. This hierarchical structure is consistent with the variance patterns revealed by the PCA, reinforcing the importance of the most representative variables for each component in morphological differentiation among groups.

#### 3.2.2 Linear discriminant analysis

The LDA model showed strong discriminatory power across the eleven groups, with an average accuracy of 96.6% and a Kappa index of 0.95, underscoring its robustness despite group imbalance. The confusion matrix (**Supplementary Table 4**) indicates perfect classification for groups C–K and *T. basilatinervium*, suggesting clear morphological differentiation. Misclassifications occurred only in Group A, where four individuals were assigned to groups E, H, I and K. These findings highlight the reliability of the LDA for groups discrimination, with minimal ambiguity restricted to Clade A.

**Figure 5:**
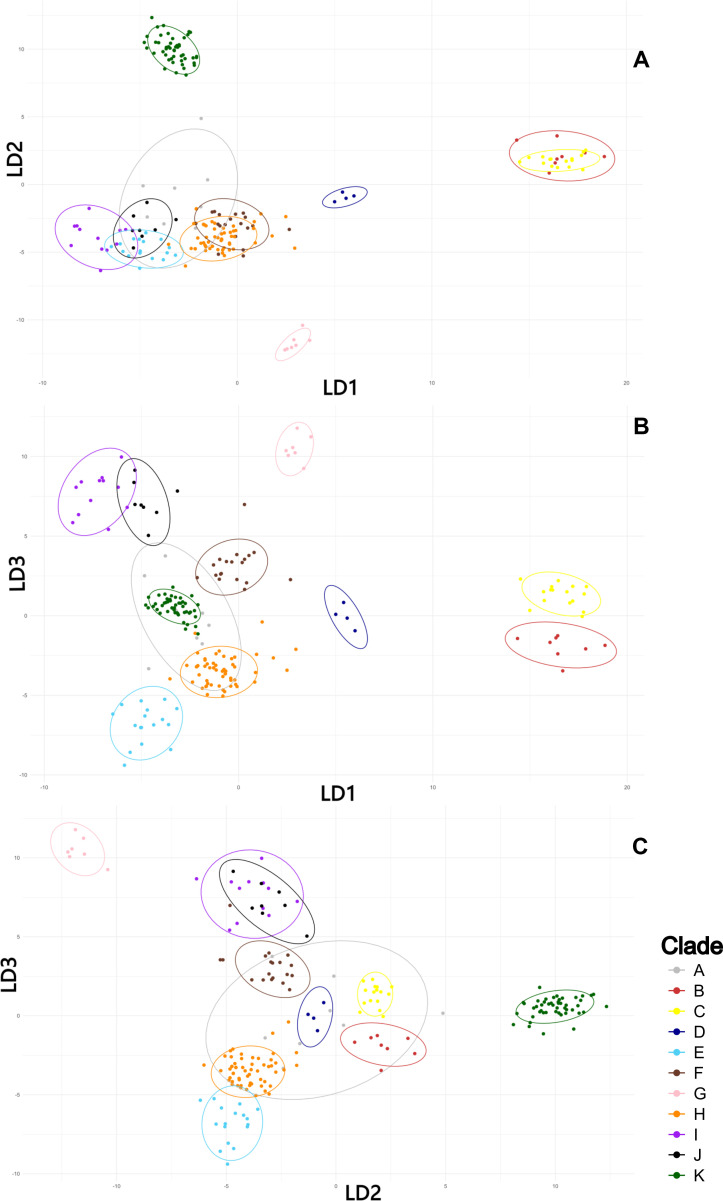
Results of the linear discriminant analysis (LDA) for South American specimens of *Trichostomum brachydontium*, based on the quantitative and qualitative data matrix. Colors represent the 11 groups defined *a priori* based on well-supported clades recovered in the molecular phylogeny (A–K). Individual specimens within each clade are shown as points. Ellipses with 95% confidence were drawn around group means. **(A)** LDA results with discriminant functions LD1 and LD2; **(B)** LDA results with LD1 and LD3; **(C)** LDA results with LD2 and LD3.

LDA plots (**Figures 5A–C**) depict the distribution of individuals across the first three discriminant functions (LD1–LD3). Contributions of the ten most influential variables to each function are shown in **Supplementary Figure S4**. For LD1, major predictors include tooth differentiation in the transition zone (47), apical termination of the leaf lamina (51), margin ornamentation (46), border extension (4), stem diameter (2), plant length (1), leaf orientation when dry (44), thickness of juxtacostal basal cell walls (63), morphology of the papillae lamina (66), and leave shape (45). LD2 is driven by border extension (4), papillae shape and number (66, 35), basal hyaline zone portion (20), width of central basal cells (27), central strand development (42), and the length of mucro, juxtacostal basal cells, and leaves (11, 28, 5). LD3 emphasizes plant length (1), basal hyaline and transition zone portions (20, 21), leaves shape (45), size and number of papillae in the middle and upper cells of the lamina (35, 40, 41, 36), and length of the leaves (5) and mucro (11).

Among these variables, eight of the thirteen most influential quantitative predictors across the first three LDs were also identified as key contributors in the PCA, underscoring the structural coherence of the dataset. Although LDA plots (**Figures 5A–C**) reveal some overlap between groups J and I and between *T. basilatinervium* and Clade C, the confusion matrix (**Supplementary Table 4**) confirms that the model successfully discriminates them. This indicates that critical information resides in dimensions beyond the first three LDs. Overall, the strong discriminative capacity of the LDA supports the interpretation that the analyzed clades represent distinct, morphologically well-defined entities, with the exception of group A.

#### 3.2.3 Hierarchical cluster analysis

**Figure 6:**
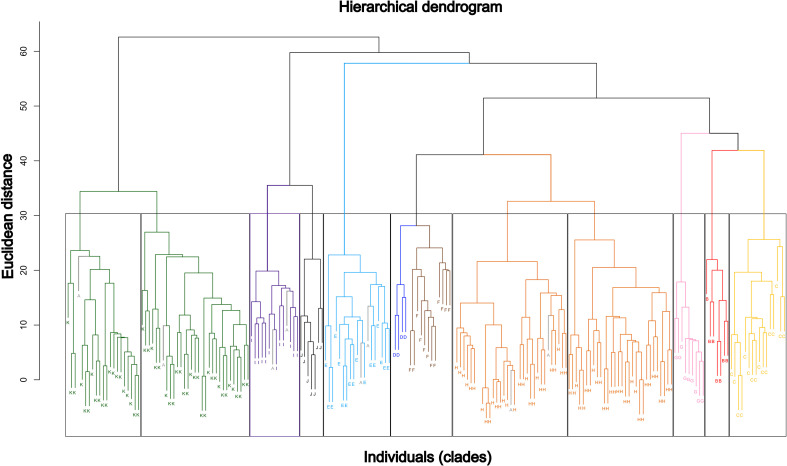
Hierarchical dendrogram obtained through a hierarchical cluster analysis (HCA) based on a combined matrix of quantitative and qualitative variables. Euclidean distance was used as the measure of similarity among individuals. The different vertical rectangles represent the clusters generated in the HCA. Colors indicate the clades identified in the molecular phylogeny A–K.

The dendrogram (**Figure 6**) identifies eleven main clusters broadly matching phylogenetic groups A–K. *Trichostomum basilatinervium*, Clades C, E, G, I, and J form cohesive cluster, indicating high intragroup homogeneity. Clades K and H split into two subclusters, suggesting internal heterogeneity or cryptic subgroups. Groups D and F cluster together despite phylogenetic separation, while Clade A shows dispersed individuals, revealing low morphological cohesion and overlap with other groups.

**Figure 7:**
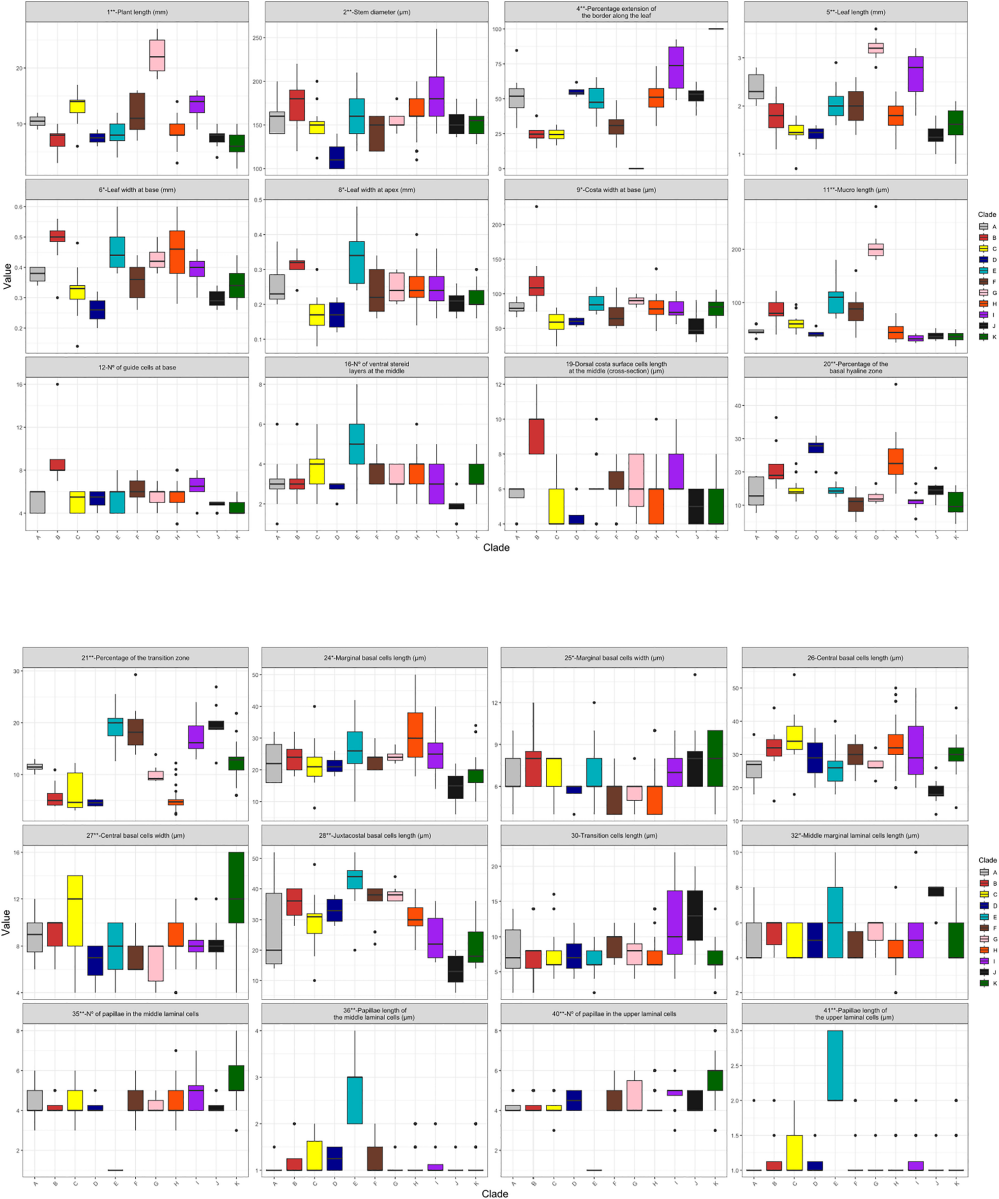
Boxplots showing the quantitative variables with the greatest discriminant capacity. Variables relevant in PCA or LDA are marked with an asterisk (*), and those relevant in both PCA and LDA with two asterisks (**). Colors indicate the 11 groups (A–K) identified in the molecular phylogeny. Boxes, 25–75 percentiles; thick horizontal line, median; vertical lines, total range excluding outliers; solid dots (•), outliers.

Box plots (**Figure 7**) illustrate variability in top discriminating variables in PCA and LDA, revealing distinct morphological patterns for most clades supported by the phylogenetic analysis.

## 4 Discussion

This study provides the first integrative assessment of the *Trichostomum brachydontium* complex in South America. Morphological and molecular evidence consistently reveal hidden diversity within the group, with ten of the eleven phylogenetically supported clades (C–K and *T. basilatinervium*) exhibiting clear morphological differentiation corroborated by multivariate analyses (LDA, PCA, HCA). The stable performance of discriminant variables across methods underscores their robustness and reliability for taxonomic delimitation.

The subfamily Trichostomoideae is confirmed as monophyletic comprising three tribes: Hyophileae, Pleuroweisieae and Trichostomeae. Within Trichostomeae, two well-supported clades were recovered: one including *Aschisma*, *Ephemerum*, *Neotrichostomum*, *Trichostomum* and *Weissia*; the other comprising *Anaschisma*, *Chionoloma*, *Eucladium*, *Pachyneuropsis*, *Pleurochaete*, *Pottiopsis*, *Streptocalypta*, *Tainoa* and *Tortella*. However, no exclusive diagnostic morphological characters were identified, and some genera were absent from the analysis, limiting taxonomic resolution. Further research with broader sampling is needed to validate this division.

The NeighborNet analyses (**Figures 2A, B**) reveal a closer relationship between Hyophileae, Pleuroweisieae and *Hydrogonium*, while Trichostomeae remains cohesive despite signs of internal subdivision. The position of *Hydrogonium* remain uncertain, as concatenated (**Figure 1**), ITS and single-marker trees (**Supplementary Figures S1**, **S2**) yield conflicting results, suggesting further evaluation.

Phylogenetic analyses consistently place specimens of *Weissia jamaicensis* within *Neotrichostomum*, rather than in *Chionoloma*, *Trichostomum* or *Weissia*. This placement is strongly supported by molecular data and coherent clustering in NeighborNet. Morphological traits (naviculate leaves, a slightly cucullate apex, and strongly differentiated along the margins basal cells) further distinguish *W. jamaicensis* from *Weissia*, which has non-naviculate leaves, a flat non-cucullate apex, and undifferentiated basal marginal cells. Additionally, *W. jamaicensis* is dioicous, whereas *Weissia* is mainly autoicous. Zander (2023) recently transferred this species to *Chionoloma* (*C. jamaicensis* (Mitt.) R.H. Zander) based on leaf morphology. However, *W. jamaicensis* differs from *Chionoloma* by its smooth rhizoids and incurved to involute leaf margins, contrasting with the papillose rhizoids (at least when young) and the usually flat, rarely incurved margins in that genus. *Neotrichostomum crispulum* is a dioicous plant with a dark-brown stem and a costa usually percurrent or very shortly excurrent, features shared with *W. jamaicensis*. Based on these combined data, we propose the new combination: *Neotrichostomum jamaicense* (Mitt.) M.J. Cano, M.T. Gallego & Omar Rodr., comb. nov. (Basionym: *Tortula jamaicensis* Mitt., J. Linn. Soc., Bot. 12: 147, 1869).

Phylogenetic analyses conducted in this study confirm that *T. involutum* belongs to the genus *Trichostomum*. Accordingly, its placement within *Trichostomum* is retained here, in contrast to Zander (2023), who transferred the species to *Chionoloma* (*Chionoloma involutum* (Sull.) R.H. Zander). As shown in the Results, *Trichostomum* is strongly supported in the concatenated phylogenetic tree, although two notable incongruences were detected. First, *T. platyphyllum* clusters with *Neotrichostomum crispulum* and *Weissia jamaicensis* in plastid gene trees and the concatenated dataset, rather than with other *Trichostomum* species. In contrast, the ITS-based tree recovers all species of the genus in a single clade (**Supplementary Figure S1**). This discordance between nuclear and plastid phylogenies may reflect contrasting inheritance patterns (uniparental in plastid DNA vs. biparental in nuclear DNA (Jankowiak et al., 2005) and processes such as hybridization or introgression, which can affect each genome differently. Additionally, plastid genomes tend to exhibit more conserved evolutionary pattern than nuclear genome, resulting in lower sequence variability (Wolfe et al., 1987; Smith, 2015; Liu et al., 2019). The second major discrepancy involves *T. brachydontium*, which splits into two well-supported clades. One includes European and Macaronesian specimens, with maximal support and close relationships to other European species such as *T. littorale*, *T. meridionale*, and *T. herzogii*. The other comprises all South American specimens, which appear more closely related to *T. involutum* (mainly distributed in the Caribbean), the Chilean *T. basilatinervium*, and *T. loxorhynchum*, a South American taxon combined recently to *Trichostomum* (Rodríguez et al., 2025). This biogeographic divergence is evident in the NeighborNet phylogenetic network generated in SplitsTree (**Figures 2A, B**), where the European and Macaronesian *T. brachydontium* clade form a distinct, well-supported group, while South American individuals are distributed across eleven clusters (including *T. basilatinervium*), indicating substantial genetic diversity. Reticulations among these groups suggests phylogenetic conflicts, likely due to hybridization, introgression, or incomplete lineage sorting (Degnan and Rosenberg, 2009; Zwickl et al., 2014) in this region.

South American clades exhibit varying degrees of morphological cohesion. Because the gametophyte remains exposed to environmental variation throughout its lifetime, it is prone to substantial plasticity and occasional convergence (Hedenäs et al., 2014), which can blur morphometric boundaries even in genetically well-supported groups. This environmentally driven variation provides a plausible explanation for the partial overlap observed in our PCA. Importantly, however, the multivariate analyses still detect meaningful structure broadly aligned with the phylogenetic lineages, indicating that despite plasticity, the morphological signal retains taxonomic relevance. Notably, Clade A shows extreme heterogeneity and phylogenetic conflict. NeighborNet reveals strong reticulation, suggesting a complex evolutionary history. Morphometrics analyses (PCA, LDA, HCA) confirm high heterogeneity, as specimens overlap with most other clades and lack a consistent morphological structure (**Figures 3A–C**). These findings indicate that Clade A does not represent a well-defined morphotype and may have a more intricate evolutionary background than phylogenetic trees alone suggest. Broader sampling is needed to resolve its complexity.

Clade B was described as *Trichostomum basilatinervium* based on detailed morphological evidence (Rodríguez et al., 2025). In the present study, this taxon is strongly supported as a distinct evolutionary lineage by both multilocus phylogenetic analyses and multivariate morphometric approaches, further corroborating its recognition at the species level.

After a direct examination of the original material of *T. hondurense* (holotype: Honduras, Santa Barbara: Mt. Santa Barbara, *B. Allen 11625*, MO 5626201!), we confirmed that the Guatemalan specimen and all other samples assigned to Clade C matches the morphology of this type specimen. Consequently, our results establish the taxonomic identity of Clade C as *T. hondurense*. This species consistently emerges as a distinct lineage closely related to *T. basilatinervium*. Despite sharing certain diagnostic features, such as weakly denticulate or crenulate margins in the transition zone and an orange-yellow KOH reaction, *T. hondurense* differs in several key traits: larger plant size, unbordered leaves (vs. basal border in *T. basilatinervium*), fewer guide cells in a single layer (vs. more in two incomplete layers), and thick-walled juxtacostal basal cells (vs. thin-walled in *T. basilatinervium*). Additionally, *T. hondurense* is characterized by an oblong-cylindrical theca and a well-developed peristome with filiform teeth (240–288 µm). *Trichostomum hondurense* was previously known only from Mesoamerica, with confirmed records in Honduras, Costa Rica, Guatemala, and Mexico (Allen, 2002; Tropicos.org, 2026). The present study provides the first evidence of the occurrence of this taxon in South America, considerably extending its previously known distribution to Chile and Venezuela.

Clade D comprises two Peruvian specimens exhibiting homogeneous morphology, which together form an independent lineage in the phylogenetic analyses, though with partial overlaps with other clades in the statistical assessments. This clade shares with *T. basilatinervium* and *T. hondurense* the presence of weakly denticulate or crenulate margins in the transition zone. However, it is distinguished by its comparatively narrower basal leaves, the smallest stem diameter among the groups examined, and the highest proportion of basal hyaline area.

Phylogenetic analyses consistently recover Clade E as a distinct lineage. Multivariate analyses identify variables related to papillae number, length, and morphology as the most discriminating. Clade E is unique in having a single pedunculate, coroniform papilla per leaf cell (2–3(4) µm high), unlike other groups that exhibit multiple non-pedicellate papillae (1–2 µm high). Clade E also shows the longest juxtacostal basal cells [(20)36–52 µm vs. 6–40(48) µm in other South American taxa]. In the HCA analysis, all individuals cluster together, reinforcing its recognition as an independent lineage.

Clade F emerges as a well-delimited lineage in the molecular analyses. In the PCA, it overlaps with most groups except Clades E and K. The LDA provides a clearer delimitation of Clade F, while the HCA reveals a close affinity with Clade D. Clades F and D share the presence of a developed basal border, obtuse or acute apices, and thin-walled juxtacostal basal cells. Nevertheless, Clade F is distinguished by its longer leaves (1.40–2.60 mm vs. 1.10–1.60 mm in Clade D), lacks denticulate margins in the transition zone, a longer mucro (34–160 µm vs. 34–56 µm in Clade D), and a flat-convex costa in cross-section, contrasting with the biconvex or circular costa in cross-section in Clade D.

Clade G is strongly supported by phylogenetic and multivariate analyses, remaining cohesive within a single HCA cluster (**Figure 6**). Although it overlaps mainly with *T. basilatinervium*, *T. hondurense* and Clades F and H in the PCA, it is clearly discriminated by LDA. This clade is distinguished by the largest plant size observed, notable leaf length (2.8–3.6 mm), and a markedly long mucro (180–220(280) µm). It also exhibits non-pedicellate coroniform papillae and lacks a differentiated border, in sharp contrast to other South American groups in which a border is always developed.

Phylogenetic analyses consistently recover Clade H as a well-supported lineage. but multivariate analyses show overlap with most clades except K, E, and J. All analyses consistently indicate affinity with Clade H. Clade F and H share a basal border, though in Clade H it typically extends to midleaf, while in Clade F this is uncommon. Differentiation is maximized in LD3, where basal hyaline and transition zones are inversely related: Clade F has a low basal hyaline zone (5–14%) and a high transition zone (14–29%), whereas Clade H has a broad basal hyaline zone [(14)20–29(46)%] and a reduced transition zone [(2)4–6(12)%]. The HCA reveals two well-defined clusters (**Figure 6**), indicating intraspecific heterogeneity without visible phenotypic differentiation. This pattern is supported by molecular analyses, which identify two clades plus two unsupported sister individuals (**Figure 1**). NeighborNet reveals two subclades with pronounced reticulation, which suggests the possibility of cryptic speciation. However, no geographical or ecological barriers are detected between these two subclades. Similar patterns have been documented in mosses (Escolástico-Ortiz et al., 2023), where genetically distinct lineages coexist despite morphological uniformity. These findings align with the chloroplast gene tree (**Supplementary Figure S2**), where subclades appear less resolved, likely due to maternal inheritance in mosses (Jankowiak et al., 2005) and the slower evolutionary rate of chloroplast DNA compared to nuclear DNA (Clegg et al., 1994), which may obscure recent divergences or reflect hybridization. Since no distinct morphotypes were identified among the examined specimens, Clade H is treated as a single lineage.

Clade I is resolved as an independent lineage in both morphological and phylogenetic analyses. In the PCA it overlaps with all groups except Clade G, while in the LDA it overlaps only with Clade J, and it forms a single cluster in HCA. Phylogenetically, Clade I is closely related to Clade E but differs mainly in the form and size of the laminal papillae (coroniform and non-pedicellate, 1–2 µm high in Clade I; vs. coroniform and pedicellate, 2–3(4) µm high in Clade E). Clade I shares papillae morphology with Clade G, yet is distinguished by a yellowish KOH reaction, a border extending to midleaf, and a transition zone of 14–21(24)%, whereas Clade G shows a yellow-orange KOH reaction, lacks a border, and has a narrower transition zone (8–11(14)%). The combined evidence from multivariate analyses and phylogenetic relationships, along with diagnostic morphological traits, justifies treating Clade I as a distinct evolutionary lineage.

Specimens assigned to Clade J are morphologically indistinguishable from *Trichostomum termitarum* (Müll. Hal.) R.H. Zander, characterized by a narrow hyaline leaf base, entire lamina, short-excurrent mucro, small, rounded cells, and gymnostomous capsules (Costa, 2016). Their morphology matches the type material of *T. termitarum* (BRASIL. Goiás, Mossamedes, *ad domicilia termitarum*, I 1893, E. Ule s.n., JE 04005068!, S B182121!, W2866!), and we therefore recognize Clade J as *T. termitarum*. Phylogenetic analyses consistently recover this species as a distinct lineage, although multivariate analyses reveal partial morphological overlap with Clade I (PCA and LDA) and occasional resemblance to young plants of Clade G. Shared traits with Clade I include a short mucro, similar basal hyaline and transition zones, yellow KOH reaction, and coroniform, non-pedicellate papillae, but Clade I differs by larger plant and leaf size and more ventral stereid layers (2–5[6] vs. 1–2[3] in *T. termitarum*). Clade G differs by lacking a midleaf border and having more ventral stereid layers (3–4 vs. fewer in *T. termitarum*). Morphological congruence with type material and molecular evidence confirm Clade J as *T. termitarum*. Despite some phenotypic overlap with Clades I and G, diagnostic traits and phylogenetic distinctiveness support its current circumscription. Previously considered endemic to Brazil (Costa, 2016), *Trichostomum termitarum* is reported here for the first time from Argentina, markedly expanding its known geographical range.

Finally, the two *T. antillarum* specimens are grouped within the Clade K, which has strong support. Morphologically, Clade K is indistinguishable from *T. antillarum*, confirming its identity. Phylogenetic analyses resolve this clade into two well-supported subclades, yet no diagnostic morphological traits have been identified, suggesting cryptic lineages, a pattern also observed in Clade H. Multivariate PCA and LDA depict *T. antillarum* as a single cohesive group with internal variability, while HCA reveals two clusters. Genetic divergence is thus uncoupled from clear morphological differentiation, potentially reflecting recent speciation or stabilizing selection, as reported in other bryophytes (McDaniel and Shaw, 2003; Kiebacher and Szövényi, 2024; Fedosov et al., 2024). In PCA space, *T. antillarum* overlaps only with Clade A and slightly with *T. basilatinervium*, *T. termitarum* and Clades I and E, due to shared traits such as mucro size (Clades I and *T. termitarum*), costa width of the superficial cells (Clade E), length of the lower basal cells and marginal median cells (*T. basilatinervium*). The most distinctive feature in *T. antillarum* is a differentiated leaf border extending to the apex, absent in other groups. Phylogenetically, *T. loxorhynchum* is closest to *T. antillarum* but differs by lacking a border and having ventrally bulging, smooth laminal cells (vs. ventrally flat and papillose in *T. antillarum*). Morphological congruence and molecular evidence confirm Clade K as *T. antillarum*. The presence of two genetic subclades without diagnostic traits highlights cryptic diversity, reinforcing the need for integrative approaches in species delimitation. Regarding the distribution of *T. antillarum*, this species is known from the Caribbean (Cuba, Guadeloupe, the US Virgin Islands, Puerto Rico and Saint Lucia), Mexico, the United States and Brazil (Zander, 2023). This study expands its known range in the Caribbean to the Dominican Republic, and in South America to Argentina, Colombia, Ecuador, Paraguay, Peru, and Venezuela.

## 5 Conclusions

Molecular and morphological evidence supports the subdivision of Trichostomeae into two well-defined groups. *Trichostomum* shows closer affinities to *Aschisma*, *Ephemerum*, *Neotrichostomum* and *Weissia* than to other genera, despite the absence of clear synapomorphies.

Phylogenetic incongruences were detected in *Hydrogonium*, which aligns with Trichostomeae in plastid data but with Hyophileae and Pleuroweisieae in nuclear data. Similarity, *Trichostomum platyphyllum* clusters with *Neotrichostomum crispulum* and *Weissia jamaicensis* in plastid analyses but remains within *Trichostomum* in nuclear analyses. *Weissia jamaicensis* is consistently placed within *Neotrichostomum*, corroborated by morphology, and a new combination is proposed. Furthermore, the position of *T. involutum* specimens within the genus *Trichostomum* is maintained.

European and Macaronesian specimens of *T. brachydontium* are clearly separated from South American representatives, which form 11 well-supported clades lacking the diagnostic traits of *T. brachydontium* sensu stricto. Accordingly, *T. brachydontium* is excluded from the South American flora. NeighborNet analyses reveal reticulation in several South American groups, suggesting hybridization, introgression, or incomplete lineage sorting.

Statistical analyses confirm phenotypic differentiation in 10 of the 11 clades, revealing strong morphological structuring consistent with their evolutionary history. Nevertheless, quantitative variables alone do not provide sufficient discriminatory power, whereas the incorporation of qualitative traits markedly enhances taxonomic resolution. In addition, the identities of Clades C, J, and K are clarified as *T. hondurense*, *T. termitarum*, and *T. antillarum*, respectively. The known distribution of *Trichostomum antillarum* is substantially expanded to Argentina, Colombia, Ecuador, Paraguay, Peru, the Dominican Republic, and Venezuela. The Central American and Mexican species *T. hondurense* is newly recorded in South America from Chile and Venezuela, while *T. termitarum* is reported for the first time from Argentina.

This study demonstrates that the integration of molecular and morphological evidence provides a robust framework for resolving species boundaries and clarifying evolutionary relationships within Trichostomeae. Our analyses reveal cryptic diversity, delimit well-supported lineages, and significantly expand the known distribution of several species in South America. These findings establish a solid basis for systematic interpretation and highlight the evolutionary complexity of the group.

Although species circumscription has been achieved here, formal nomenclatural acts and detailed taxonomic descriptions remain pending. A forthcoming contribution will address all delimited species by assigning valid names, stabilizing synonymy, and providing comprehensive morphological diagnoses and identification tools. This integrative taxonomic treatment will consolidate the classification of Trichostomeae and facilitate its application in ecological and evolutionary research.

## Data Availability

The datasets presented in this study can be found in online repositories. The names of the repository/repositories and accession number(s) can be found in the article/**Supplementary Material**.
