# Supplementary material for: Hidden diversity in the Trichostomum brachydontium complex (Pottiaceae, Bryophyta) revealed by integrative taxonomy
Source: Front Plant Sci. 2026 Apr 21;17:1822444. doi: 10.3389/fpls.2026.1822444 (PMC13139172; doi:10.3389/fpls.2026.1822444)
Supplement: Supplementary file 1 [file SupplementaryFile1.zip › Supplementary_material/Supplementary_FIGURE_S1.docx]

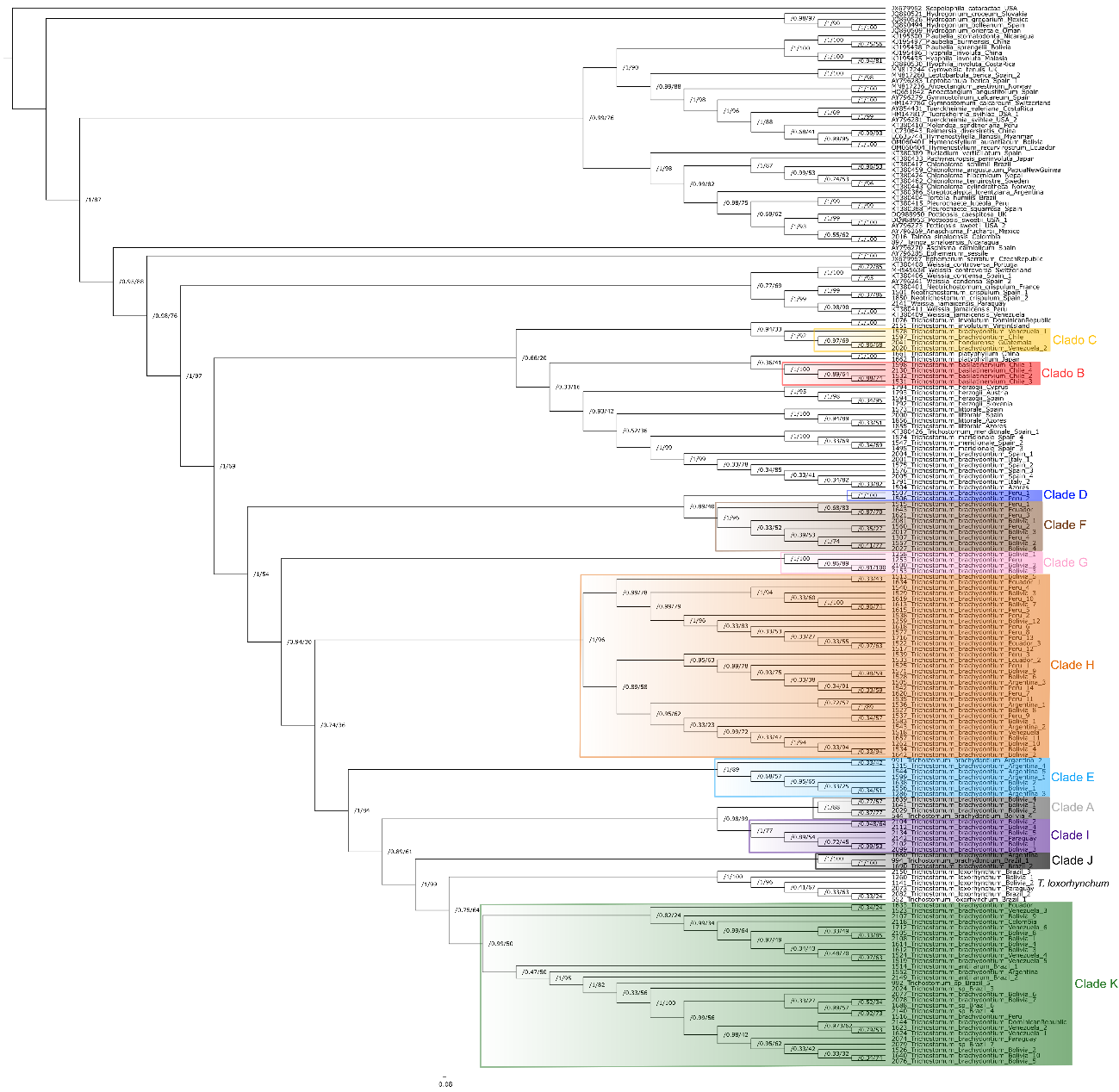


**FIGURE S1** ML tree inferred from the nuclear dataset in IQ-TREE v.2.3.4, showing the phylogenetic relationships among all specimens included in the study. Numbers at the nodes (from left to right) indicate aBayes and UFBoot (%) support values.
