# Supplementary material for: Hidden diversity in the Trichostomum brachydontium complex (Pottiaceae, Bryophyta) revealed by integrative taxonomy
Source: Front Plant Sci. 2026 Apr 21;17:1822444. doi: 10.3389/fpls.2026.1822444 (PMC13139172; doi:10.3389/fpls.2026.1822444)
Supplement: Supplementary file 1 [file SupplementaryFile1.zip › Supplementary_material/Supplementary_FIGURE_S3.docx]

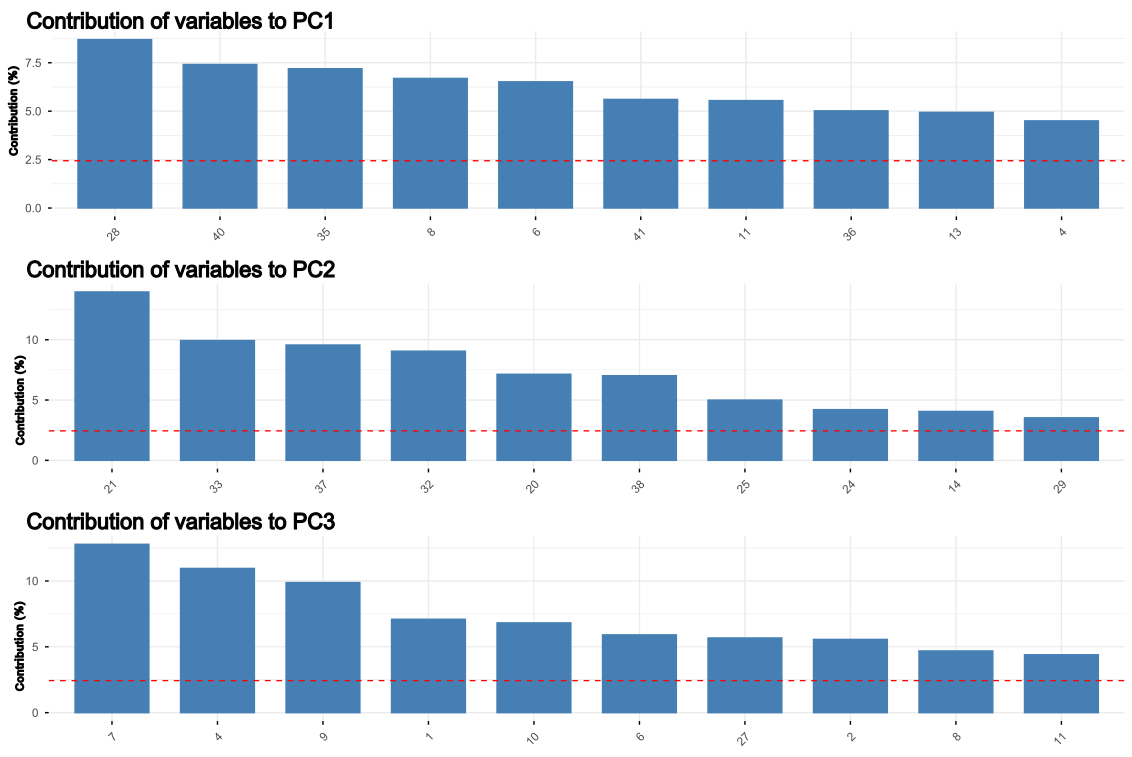


**FIGURE S3** Percentage of influence (percent contribution) of the 10 most influential variables in each of the first three principal components (PC1, PC2, and PC3). The red line indicates the group mean. Contribution represents the relative weight of each variable in defining each component.
