# Supplementary material for: Hidden diversity in the Trichostomum brachydontium complex (Pottiaceae, Bryophyta) revealed by integrative taxonomy
Source: Front Plant Sci. 2026 Apr 21;17:1822444. doi: 10.3389/fpls.2026.1822444 (PMC13139172; doi:10.3389/fpls.2026.1822444)
Supplement: Supplementary file 1 [file SupplementaryFile1.zip › Supplementary_material/Supplementary_FIGURE_S4.docx]

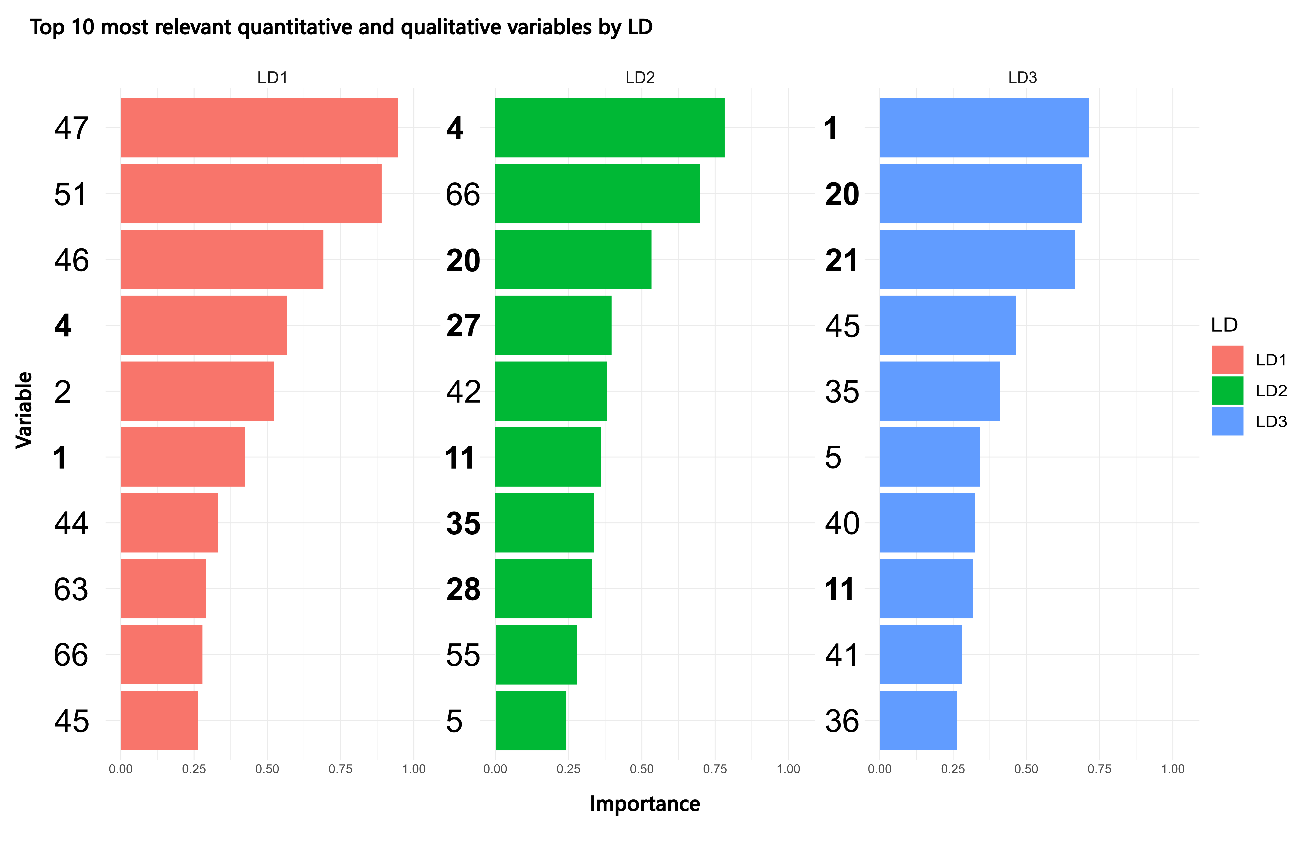


**FIGURE S4** Bar chart representation of the 10 variables with the greatest influence on each of the discriminant functions LD1, LD2, and LD3. The variables were selected independently for each function, according to the discriminant coefficients obtained through LDA analysis. Quantitative variables with a dominant contribution to the PCA are highlighted in bold.
