# Supplementary material for: Hidden diversity in the Trichostomum brachydontium complex (Pottiaceae, Bryophyta) revealed by integrative taxonomy
Source: Front Plant Sci. 2026 Apr 21;17:1822444. doi: 10.3389/fpls.2026.1822444 (PMC13139172; doi:10.3389/fpls.2026.1822444)
Supplement: Supplementary file 1 [file SupplementaryFile1.zip › Supplementary_material/Supplementary_TABLE S1.docx]

**TABLE S1** Vouchers and GenBank accession numbers for taxa used in the molecular phylogenetic analysis. Taxon name, country, largest political subdivision, collector(s) and collector number(s), herbarium abbreviation, GenBank accession number for the *atp*B-*rbc*L, *trn*G, *trn*L-F, *rps*4 and nrITS. For new sequences, the own number assigned for molecular analysis is provided in parentheses, with the GenBank accession number highlighted in bold; ‘-’ indicates missing data.

*Anaschisma fruchartii* (Müll. Hal.) R.H. Zander, G.M. Suárez & M.S. Jiménez, Mexico, Guanajuato, *Delgadillo s.n.* (NY), -, -, -, -, AY796269. *Anoectangium angustifolium* Mitt., Spain, Canary Island, *Köckinger s.n.* (CBFS), -, -, -, -, HQ651842. *Anoectangium aestivum* (Hedw.) Spruce, Norway, Tromsø, *Kučera 15892* (CBFS), -, -, -, -, MN817236. *Aschisma carniolicum* (F. Weber & D. Mohr) Lindb., Spain, Huelva, *Cano et al. s.n.* (MUB), -, -, -, -, AY796270. *Chionoloma angustatum* (Mitt.) M. Menzel, Papua New Guinea, Morobe, *Norris 62286* (S), KT380229, KT380302, KT380381, -, KT380459. *Chionoloma daldinianum* (De Not.) M. Alonso, M.J. Cano & J.A. Jiménez, Norway, Rogaland, *Long 38149* (E), KT380214, KT380288, KT380367, -, KT380443**.** *Chionoloma hibernicum* (Mitt.) M. Alonso, M.J. Cano & J.A. Jiménez, Nepal, Bagmati, *Long 30538* (E), KT380196, KT380271, KT380349, -, KT380424. *Chionoloma schlimii* (Müll. Hal.) M. Alonso, M.J. Cano & J.A. Jiménez, Brazil, Goiás, *Soares et al. 2258* (MUB), KT380191, KT380266, KT380342, -, KT380417. *Ephemerum serratum* (Schreb. ex Hedw.) Hampe, Czech Republic, Velka nad Velickou, *Košnar 692* (CBFS), -, -, -, JX679985, JX679967. *Ephemerum sessile* (Bruch) Müll. Hal., location and collector unknown, (MUB) -, -, -, -, AY796285. *Eucladium verticillatum* (With.) Bruch & Schimp., Spain, Cantabria, *Cano 6702* (MUB), KT380164, KT380236, KT380310, -, KT380389. *Gymnostomum calcareum* Nees & Hornsch., Spain, Cádiz, *Guerra et al. s.n.* (MUB), -, -, -, -, AY796279; Switzerland, Muzzano, *Kučera* 6508 (CBFS), -, -, -, HM147786, HM147812. *Gyroweisia tenuis* (Hedw.) Schimp., United Kingdom, Derbyshire, *Blockeel 42/036*, -, -, -, -, MN817244. *Hydrogonium bolleanum* (Müll. Hal.) A. Jaeger, Spain, Murcia, *Kučera 13670* (CBFS), -, -, -, JX679970, JQ890494. *Hydrogonium croceum* (Brid.) Jan Kučera, Slovakia, Motycky, *Kučera 1087* (CBFS), -, -, -, JQ890462, JQ890521. *Hydrogonium gregarium* (Mitt.) Jan Kučera, Mexico, Guerrero, *Eckel 188986* (DUKE), -, -, -, JQ890467, JQ890526. *Hydrogonium orientale* (F. Weber) Jan Kučera, Oman, Wadi Tiwi, *Rothfels 2763* (DUKE), -, -, -, JQ890449, JQ890509. *Hymenostyliella llanosii* (Müll. Hal.) H. Rob*.*, Myanmar, *Inoue 7547* (TNS), Myanmar, Kayah State, *Inoue 7547* (TNS), -, -, -, LC635742, LC635744. *Hymenostylium aurantiacum* Mitt., Bolivia, Tarija, *Churchill et al.* *23336* (MUB), -, -, -, -, OM060401. *Hymenostylium recurvirostrum* (Hedw.) Dixon, Ecuador, Azuay, *Cano & Gallego* *2905a* (MUB), -, -, -, OL963738, OM060404. *Hyophila involuta* (Hook.) A. Jaeger, China, Yunnan, *Zhang 9797* (SZG), -, -, -, KJ195510, KJ195496; Costa Rica, Barra Honda, *Hauer s.n*. (CBFS), -, -, -, JQ890477, JQ890530; Malaysia, Penang*, Zhang 8636* (SZG), -, -, -, KJ195508, KJ195495. *Leptobarbula berica* (De Not.) Schimp., Spain (1), Balearic Island, *Cano 1014* (MUB), -, -, -, -, AY796283; Spain (2), Murcia, *Kučera 13640* (CBFS), -, -, -, -, MN817260. *Molendoa sendtneriana* (Bruch & Schimp.) Limpr., Peru, Departamento La Libertad, *Cano & Jiménez 5333* (MUB), KT380185, KT380259, KT380335, -, KT380410. *Neotrichostomum crispulum* (Bruch) R.H. Zander, Spain (1), Málaga, *B. Cabezudo s.n.* (MUB), (1501) **PX711831**, **PX898007**, **PX872771**, **PX704529**, **PZ069055**; Spain (2), Canay Island, *Cano 8816* (MUB), (1850) **PX711911**, -, -, -, **PZ069058**; France, Aquitaine, *Cano 6521* (MUB), KT380176, KT380248, KT380324, -, KT380401. *Neotrichostomum jamaicense* (Mitt.) M.J. Cano, M.T. Gallego & Omar Rodr., Paraguay, Departamento Guaira, *E. Bordas 199* (MUB), (2141) **PX711889**, -, **PX872772**, **PX704478**, **PZ069129**; Peru, Departamento Cajamarca, *Cano & Jiménez 5174* (MUB), KT380186, KT380260, KT380336, -, KT380411; Venezuela, Lara, *Grande et al. 5764* (MUB), KT380184, KT380258, KT380334, -, KT380409. *Pachyneuropsis perinvoluta* (Tixier) M. Alonso, M.J. Cano & J.A. Jiménez, Japan, Kyushu, *Yamaguchi 24446* (HIRO), KT380205, KT380279, KT380358, -, KT380433. *Plaubelia burmensis* (B.C. Tan & Z. Iwats.) S. He & Li Zhang, China, Yunnan, *Mao 101* (HIRO), -, -, -, KJ195511, KJ195497. *Plaubelia sprengelii* (Schwägr.) R.H. Zander, Bolivia, Tarija, *Linneo & Nee LN203* (HIRO), -, -, -, -, KJ195498. *Plaubelia stomatodonta* (Cardot) R.H. Zander, Nicaragua, Esteli, *Zhang 9797* (HIRO), -, -, -, KJ195512, KJ195500. *Pleurochaete squarrosa* (Brid.) Lindb., Spain, Balearic Island, *Cano et al. s.n.* (MUB), KT380163, GU953705, GU953730, -, KT380388; Peru, Departamento Cajamarca, *Cano et al. 5003* (MUB), KT380189, KT380264, KT380340, -, KT380415. *Pottiopsis caespitosa* (Bruch ex Brid.) Blockeel & A.J.E. Sm., Spain, Gerona, *Corominas s.n.* (BCB), -, -, -, -, DQ988950. *Pottiopsis sweetii* (E.B.Bartram) Ros & O. Werner, USA (1), Nuevo Mexico, *Castetter & Stark NV-520* (UNLV), -, -, -, -, DQ988953; USA (2), Nevada, *Stark NV-143B* (UNLV), -, -, -, -, AY796273. *Reimersia diversiretis* (Broth. ex Hand.-Mazz.) Shevock, W.Z. Ma, S. He & D.G. Long, China, Yunnan, *Shevock & Ma 50685* (TNS), -, -, -, LC730645, LC730643. *Scopelophila cataractae* (Mitt.) Broth., USA, North Carolina, *Shaw s.n.* (CBFS), -, -, -, JX679983, JX679962. *Streptocalypta lorentziana* Müll. Hal., Argentina, Tucumán, *Cano et al. 4181* (MUB), KT380162, KT380233, KT380307, -, KT380386. *Tainoa sinaloensis* (E.B. Bartram) R.H. Zander, Colombia, Magdalena, *Churchill & Linares 18381* (MO), (2016) -, -, -, -, **PZ069135**; Nicaragua, Madriz, *Cano & Alonso 9699* (MUB), (897) **PX711909**, **PX898004**, **PX872863**, -, **PZ069095**. *Tortella humilis* (Hedw.) Jenn., Brazil, Minas Gerais, *Soares et al. 1728* (MUB), KT380179, KT380252, KT380328, -, KT380404. *Trichostomum sp*. (Clade A), Bolivia (1), Chuquisaca, *Lozano & Flores 2787* (MO), (1641), **PX711918**, -, **PX872836**, -, **PZ069073**; Bolivia (2), Chuquisaca, *Lozano 1059* (MO), (2029), -, -, **PX872810**, **PX704541**, **PZ069074**; Bolivia (3), Cochabamba, *Linneo 2222* (MUB), (544), -, -, **PX872811**, -, **PZ069133**; Bolivia (4), Tarija, *Churchill et al. 23307* (MO), (1639) **PX711903**, -, **PX872809**, -, **PZ069072**. *Trichostomum sp*. (Clade D), Peru (1), Departamento Cajamarca, *Cano et al. 5136* (MUB), (1507) **PX711844**, **PX898015**, **PX872785**, **PX704542**, **PZ069008**; Peru (2), Departamento Piura, *Cano et al. 5239* (MUB), (1506) **PX711845**, **PX898014**, **PX872786**, **PX704532**, **PZ069037.** *Trichostomum sp*. (Clade E), Argentina (1), Córdoba, *Cano & Jiménez 3960* (MUB), (1599) **PX711893**, **PX898053**, **PX872859**, **PX704539**, **PZ069025**; Argentina (2), Jujuy, *Cano & Alonso 8452* (MUB), (991) **PX711921**, -, **PX872837**, -, **PZ069103**; Argentina (3), Tucumán, *Cano et al. 4184* (MUB), (1286) -, -, -, -, **PZ069019**; Argentina (4), Tucumán, *Cano et al. 4020* (MUB), (1315) -, -, -, **PX704544**, **PZ069020**; Argentina (5), Tucumán, *Cano et al. 4036c* (MUB), (1544) **PX711848**, **PX898000**, **PX872787**, -, **PZ069022**; Bolivia (1), Chuquisaca, *Cano et al. 3596b* (MUB), (1556) **PX711904**, **PX898038**, **PX872851**, -, **PZ069023**; Bolivia (2), Tarija, *Churchill et al. 23271* (MO), (1638) **PX711878**, -, **PX872835**, -, **PZ069028**. *Trichostomum* *sp.* (Clade F), Bolivia (1), Chuquisaca, *Lozano & Lliully 2397* (MO), (2081) -, -, -, -, **PZ069136**; Bolivia (2), Santa Cruz, *Churchill & Linneo 24564* (MUB), (1557) **PX711880**, **PX898048**, **PX872815**, -, **PZ069119**; Bolivia (3), Santa Cruz, *Churchill 20996* (MO), (2017) **PX711851**, **PX897984**, -, -, **PZ069131**; Bolivia (4), Santa Cruz, *Churchill et al. 23885* (MO), (2027) **PX711853**, **PX897985**, **PX872790**, **PX704489**, **PZ069122**; Ecuador, Pichincha, *Toapanta & Quishpe 300* (MO), (1643) **PX711850**, -, **PX872849**, -, **PZ069052**; Peru (1), Ancash, *Cano & Alonso 7116* (MUB), (1515) **PX711849**, **PX897976**, **PX872768**, **PX704488**, **PZ069012**; Peru (2), Departamento Cajamarca, *Cano & Jiménez 5179* (MUB), (1560) **PX711876**, -, **PX872788**, -, **PZ069061**; Peru (3), Departamento Junín, *Cano & Guerra 2089* (MUB), (1621) **PX711894**, **PX898042**, **PX872865**, -, **PZ069042**; Peru (4), Lima, *Cano et al. 7645* (MUB), (1307) **PX711852**, **PX897977**, **PX872789**, -, **PZ069050**. *Trichostomum sp.* (Clade G), Bolivia (1), La Paz, *Fuentes et al. 10832* (MO), (1258) **PX711910**, -, **PX872866**, **PX704523**, **PZ069076**; Bolivia (2), La Paz, *Fuentes et al. 10760a* (MO), (2100) -, -, -, -, **PZ069039**; Bolivia (3), La Paz, *Fuentes et al.* *10760b* (MO), (2153) -, -, -, -, **PZ069040**; Peru, Departamento Cajamarca, *Cano et al. 4995* (MUB), (1253) **PX711905**, **PX898041**, **PX872854**, -, **PZ069085**. *Trichostomum sp*. (Clade H), Argentina (1), Córdoba, *E. Fuertes s.n.* (MUB), (1536) **PX711855**, -, -, **PX704491**, **PZ069045**; Argentina (2), Córdoba, *Cano & Jiménez 2975* (MUB), (1543) **PX711916**, **PX897990**, **PX872799**, **PX704511**, **PZ069032**; Argentina (3), Tucumán, *Cano et al. 4220* (MUB), (1505) **PX830579**, **PX898013**, **PX872766**, **PX704533**, **PZ069009**; Bolivia (1), Chuquisaca, *Cano et al. 3605* (MUB), (1583) -, -, **PX872801**, -, **PZ069010**; Bolivia (2), Chuquisaca, *Lozano, et al. 2831* (MO), (1642) -, -, **PX872847**, **PX704496**, **PZ069057**; Bolivia (3), Cochabamba, *Cano et al. 3424* (MUB), (1529) **PX711882**, **PX898027**, **PX872858**, **PX704521**, **PZ069051**; Bolivia (4), Cochabamba, *Cano et al. 3444* (MUB), (1534) **PX711859**, -, **PX872841**, **PX704492**, **PZ069077**; Bolivia (5), La Paz, *Cano et al. 3892* (MUB), (1513) **PX711854**, **PX898001**, **PX872791**, **PX704490**, **PZ069031**; Bolivia (6), La Paz, *Cano & Jiménez 3813* (MUB), (1528) **PX711923**, **PX897996**, **PX872804**, **PX704519**, **PZ069062**; Bolivia (7), La Paz, *Cano & Jiménez 3799* (MUB), (1613) -, **PX898051**, -, -, **PZ069081**; Bolivia (8), Oruro, *Cano et al. 3371* (MUB), (1527) **PX711907**, **PX898035**, **PX872843**, **PX704536**, **PZ069013**; Bolivia (9), Oruro, *Cano et al. 3389b* (MUB), (1571) **PX711860**, **PX898002**, **PX872802**, **PX704540**, **PZ069116**; Bolivia (10), Santa Cruz, *Carreño & Linneo 523a* (MUB), (1262) -, -, -, -, **PZ069029**; Bolivia (11), Santa Cruz, *Carreño & Linneo 523b* (MUB), (1657) **PX711922**, **PX898023**, **PX872808**, -, **PZ069034**; Bolivia (12) Santa Cruz, *Sanjinés et al. 3024* (MO), (1259) **PX711902**, **PX898052**, **PX872794**, -, **PZ069018**; Ecuador (1), Azuay, *Cano et al. 2832a* (MUB), (1634) **PX711896**, **PX898022**, **PX872840**, -, **PZ069027**; Ecuador (2), Carchi, *Cano 3204* (MUB), (1533) **PX711885**, -, **PX872806**, **PX704520**, **PZ069030**; Ecuador (3), Chimborazo, *Cano 3132* (MUB), (1522) **PX711856**, **PX897989**, **PX872850**, **PX704524**, **PZ069117**; Peru (1), Ancash, *Cano et al. 7106b* (MUB), (1525) **PX711884**, -, **PX872805**, **PX704530**, **PZ069017**; Peru (2), Ancash, *Cano et al. 7069* (MUB), (1538) -, -, **PX872830**, **PX704535**, **PZ069075**; Peru (3), Ancash, *Cano et al. 7095* (MUB), (1539) -, **PX898037**, **PX872842**, **PX704514**, **PZ069060**; Peru (4), Ancash, *Cano et al. 7120* (MUB), (1540) **PX711883**, **PX898008**, **PX872795**, **PX704518**, **PZ069046**; Peru (5), Ancash, *Cano et al. 7096* (MUB), (1615) **PX711881**, **PX898045**, **PX872848**, -, **PZ069068**; Peru (6), Ancash, *Cano et al. 6884a* (MUB), (1618) -, **PX898044**, **PX872833**, -, **PZ069026**; Peru (7), Ancash, *Cano et al. 7050* (MUB), (1620) **PX711886**, **PX898047**, **PX872807**, -, **PZ069033**; Peru (8), Departamento Cajamarca, *Cano et al. 5014* (MUB), (1577) **PX830580**, -, **PX872769**, -, **PZ069024**; Peru (9), Departamento La Libertad, *Cano & Jiménez 5364* (MUB), (1537) **PX711875**, **PX897995**, **PX872800**, **PX704494**, **PZ069053**; Peru (10), Departamento La Libertad, *Cano et al. 4981b* (MUB), (1619) **PX711895**, **PX898046**, **PX872793**, -, **PZ069078**; Peru (11), Departamento Puno, *Cano 2274* (MUB), (1535) **PX711914**, **PX897994**, **PX872798**, **PX704493**, **PZ069082**; Peru (12), Huánuco, *Cano et al. 7333a* (MUB), (1517) **PX711857**, **PX897999**, **PX872796**, **PX704516**, **PZ069021**; Peru (13), Huánuco, *Cano et al. 7374a* (MUB), (1716) -, -, -, -, **PZ069054**; Peru (14), Lima, *Cano et al. 7576a* (MUB), (1542) -, -, **PX872803**, **PX704495**, **PZ069035**; Venezuela, Mérida, *Grande et al. 5965* (MUB), (1518) **PX711858**, **PX897998**, **PX872797**, **PX704537**, **PZ069124**. *Trichostomum sp.* (Clade I), Bolivia (1), Chuquisaca, *R. Lozano 1119* (MO), (2102) -, -, -, -, **PZ069064**; Bolivia (2), Santa Cruz, *Churchill et al. 21670* (MO), (2104) -, -, -, **PX704497**, **PZ069080**; Bolivia (3), Tarija, *Churchill et al. 23334* (MO), (2099) -, -, -, **PX704498**, **PZ069059**; Bolivia (4), Tarija, *Apaza et al. 59* (MO), (2112) -, -, **PX872770**, **PX704528**, **PZ069084**; Bolivia (5), Tarija, *Linneo et al. 3777* (MUB), (2134) -, -, -, -, **PZ069071**; Paraguay, Departamento Amambay, *William R. Buck 12501* (MUB), (2142) -, -, -, -, **PZ069070.** *Trichostomum antillarum* R.H. Zander, Argentina, Salta, *Cano & Alonso 8507* (MUB), (1552) **PX711898**, **PX898033**, **PX872861**, -, **PZ069113**; Bolivia (1), La Paz, *Cayola* 1536 (MO), (2108) -, -, **PX872792**, **PX704505**, **PZ069110**; Bolivia (2), Santa Cruz, *Churchill 22248* (MUB), (1526) **PX711919**, -, **PX872823**, **PX704527**, **PZ069130**; Bolivia (3), Santa Cruz, *Cano et al. 3554* (MUB), (1612) **PX711915**, **PX898029**, **PX872862**, -, **PZ069127**; Bolivia (4), Santa Cruz, *Churchill et al. 23032* (MUB), (1614) **PX830581**, **PX898036**, **PX872828**, -, **PZ069112**; Bolivia (5), Santa Cruz, *Sanjinés 3024* (MUB), (2076) **PX711872**, **PX897992**, -, **PX704509**, **PZ069134**; Bolivia (6), Santa Cruz, *Sanjinés 3025* (MO), (2077) **PX711868**, **PX897978**, **PX872820**, **PX704506**, **PZ069098**; Bolivia (7), Santa Cruz, *A. Fuentes 292-A* (MO), (2078) -, -, -, -, **PZ069132**; Bolivia (8), Santa Cruz, *Churchill 22501* (MO), (2105) -, -, -, -, **PZ069109**; Bolivia (9), Santa Cruz, *Churchill* *22730* (MO), (2107) -, -, -, -, **PZ069107**; Bolivia (10), Tarija, *S. Abrahamczyk 7* (MO), (1640) **PX711891**, -, **PX872852**, -, **PZ069126**; Brazil (1), Matto Grosso, *Athayde Filho s.n.* (MUB), (1514) **PX711874**, **PX898016**, **PX872827**, **PX704510**, PV053390; Brazil (2), Minas Gerais, *Câmara 2163* (MUB), (2149) -, -, -, -, PV053391; Brazil (3), Bahía, *B. Boom & S. Mori 1061* (MO), (2024) -, -, -, -, **PZ069104**; Brazil (4), Bahía, *Daniel M. Vital & William R. Buck 20368a* (MUB), (2140) -, -, -, -, **PZ069097**; Brazil (5), Minas Gerais, *Soares et al. 2092* (MUB), (992) **PX711908**, **PX898019**, **PX872819**, **PX704534**, **PZ069087**; Brazil (6), Minas Gerais, *Câmara 2164* (MUB), (1688) **PX711869**, **PX897975**, -, **PX704507**, **PZ069096**; Brazil (7), Pernambuco, *Katia C. Porto 823* (MO), (2079) **PX711873**, -, **PX872824**, -, **PZ069099**; Colombia, Caldas, *Churchill & Arbeláez 15629* (MO), (2118) -, -, -, -, **PZ069128**; Ecuador, Azuay, *Cano et al. 2890* (MUB), (1633) **PX711866**, -, **PX872818**, -, **PZ069108**; Paraguay, Alto Paraguay, *Churchill & Florentín 20159b* (MUB), (2074) -, **PX898032**, -, -, **PZ069102**; Peru, Departamento Cajamarca, *Cano & Jiménez 5175* (MUB), (1516) **PX711870**, **PX898025**, **PX872845**, **PX704508**, **PZ069100**; Dominican Republic, Puerto Plata, *William R. Buck 19173* (MUB), (2144) -, -, -, -, **PZ069091**; Venezuela (1), Distrito Capital, *Grande et al. 5717* (MUB), (1624) **PX711899**, -, **PX872822**, -, **PZ069090**; Venezuela (2), Lara, *Grande et al. 5740* (MUB), (1623) **PX711871**, -, **PX872821**, **PX704538**, **PZ069089**; Venezuela (3), Mérida, *Grande et al. 5952b* (MUB), (1523) **PX711920**, **PX898017**, **PX872855**, **PX704525**, **PZ069106**; Venezuela (4), Mérida, *Grande et al. 5948* (MUB), (1524) **PX711888**, -, **PX872844**, **PX704526**, **PZ069123**; Venezuela (5), Táchira, *Grande et al. 6035* (MUB), (1519) **PX711867**, **PX897991**, **PX872846**, **PX704513**, **PZ069105**; Venezuela (6), Trujillo, *Grande et al. 5848* (MUB), (1712) **PX711865**, -, -, **PX704504**, **PZ069101**. *Trichostomum basilatinervium* M.J. Cano, M.T. Gallego & Omar Rodr., Chile (1), Región del Libertador Bernardo O'Higgins, *J. Larraín 31684* (MUB), (1598) **PX711843**, **PX898043**, **PX872814**, -, **PZ069088**; Chile (2), Región de Maule, *J. Larraín 28634* (MUB), (1532) **PX711842**, **PX897997**, **PX872832**, **PX704522**, **PZ069086**; Chile (3), Región de Valparaíso, *J. Larraín 40620* (MUB), (1531) **PX711841**, **PX897993**, **PX872813**, **PX704486**, **PZ069118**; Chile (4), Región de Valparaíso, *J. Larraín 40633* (MUB), (2130) -, -, -, -, **PZ069125**. *Trichostomum brachydontium* Bruch, Spain (1), Cádiz, *Cano 4775* (MUB), (2004) **PX711833**, **PX897979**, **PX872775**, **PX704480**, **PZ069092**; Spain (2), Málaga, *B. Cabezudo s.n.* (MUB), (1575), **PX711832**, **PX898011**, **PX872860**, **PX704479**, **PZ069120**; Spain (3), Málaga, *B. Cabezudo s.n.* (MUB), (1576), **PX711913**, **PX898010**, **PX872773**, **PX704512**, **PZ069121**; Spain (4), Cádiz, *Guerra et al. s.n.* (MUB), (2005), **PX711834**, **PX897980**, **PX872776**, **PX704481**, **PZ069056**; Italy (1), Puglia, *Cano 8887* (MUB), (2001), **PX711835**, **PX897981**, **PX872856**, **PX704482**, **PZ069016**; Italy (2), Campania, *Cano* *8926* (MUB),(1791) -, **PX898049**, **PX872774**, **PX704515**, **PZ069047**; Portugal, Azores, *Cano 6696* (MUB), (1504) **PX711877**, **PX898040**, **PX872777**, **PX704543**, **PZ069011**. *Trichostomum herzogii* Ros, O. Werner & R.D. Porley, Austria, Vorarlberg, *Cano 10427* (MUB), (1793) **PX711892**, **PX898024**, **PX872780**, **PX704485**, **PZ069049**; Cyprus, Famagusta, *Cano 10679* (MUB); (1794) **PX711900**, **PX898009**, **PX872831**, -, **PZ069043**; Slovenia, Radovljica, *Cano 10153* (MUB), (1792), **PX711912**, **PX898050**, **PX872829**, -, **PZ069048**; Spain, Cantabria, *Cano 10488a* (MUB), (1594) **PX711901**, **PX898031**, **PX872838**, -, **PZ069041**. *Trichostomum* *hondurense* B.H. Allen, Chile, Región de Magallanes, *P. Drapela 823* (MUB), (1597) **PX711847**, **PX898020**, **PX872834**, -, **PZ069036**; Guatemala, Huehuetenango, *Eve Laeger et al. 3674* (MO), (2041) **PX711846**, **PX897988**, -, **PX704487**, **PZ069038**; Venezuela (1), Distrito Capital, *Grande et al. 5733* (MUB), (1578) **PX711917**, **PX898039**, **PX872857**, -, **PZ069063**; Venezuela (2), Trujillo, *Dana Griffin, III & Manuel López F. PV-1241* (MO), (2020) -, -, -, -, **PZ069093**. *Trichostomum involutum* Sull., Dominican Republic, La Vega, *Croat 97751* (MUB) (1076), -, -, -, -, **PZ069014**; USA, Virgin Islands, San Juan, *B. Allen 31822* (MO), (2151) -, -, -, -, **PZ069015**. *Trichostomum littorale* Mitt., Spain (1), Málaga, *B. Cabezudo s.n.* (MUB), (1573), **PX711839**, **PX898012**, **PX872782**, **PX704531**, **PZ069111**; Spain (2), Madrid, *O. Rodríguez s.n.* (MUB), (2000) **PX711879**, -, **PX872784**, -, **PZ069044**; Portugal (1), Azores, *Cano 6656* (MUB), (1855), **PX711840**, **PX898006**, **PX872783**, -, **PZ069079**; Portugal (2), Azores, *Cano 1721b* (MUB), (1856), -, **PX898018**, **PX872781**, -, **PZ069069**; *Trichostomum loxorhynchum* (Müll. Hal. ex Ångstr.) M.J. Cano, M.T. Gallego & Omar Rodr., Brazil (1), Distrito Federal, *Soares et al. 2214* (MUB), (552), **PX711887**, **PX898003**, **PX872825**, -, PV053394; Brazil (2), Bahía, *Peralta & Perez-Maluf 10291* (MUB), (2082) **PX711861**, **PX898021**, -, -, PV053396; Brazil (3), Bahía, *Daniel M. Vital & William R. Buck 20368b* (MUB), (2150) -, -, -, **PX704500**, **PZ069083**; Bolivia (1), Santa Cruz, *Linneo & Villarroel 3830* (MUB), (1260), **PX711897**, -, **PX872853**, -, PV053393; Bolivia (2), Santa Cruz, *Linneo & Villarroel 3792* (MUB), -, -, -, -, PV053392; Paraguay, Alto Paraguay, *Churchill & Florentín 20159a* (MUB), (2073), **PX711862**, **PX898005**, **PX872826**, **PX704499**, PV053395. *Trichostomum meridionale* Ros, O. Werner & R.D. Porley, Spain (1), Murcia, *Cano 6745* (MUB), KT380199, -, -, -, KT380426; Spain (2), Murcia, *Cano 10894* (MUB) (1547), **PX711836**, **PX897982**, **PX872778**, **PX704483**, **PZ069066**; Spain (3), Málaga, *B. Cabezudo s.n.* (MUB), (1495), **PX711837**, **PX897983**, **PX872767**, **PX704484**, **PZ069065**; Spain (4), Murcia, *Cano 10833* (MUB), (1574), **PX711838**, **PX898034**, **PX872779**, **PX704517**, **PZ069067**. *Trichostomum platyphyllum* (Broth. ex Ihsiba) P.C. Chen, China, Nantou County, *Shevock & Yang 41653* (MUB), (1661) **PX711906**, **PX898026**, **PX872839**, -, PV053389; Japan, Chugoku, *Orgaz s.n.* (MUB), (1662), **PX711890**, **PX898028**, **PX872864**, -, PV053388. *Trichostomum termitarum* (Müll. Hal.) R.H. Zander*,* Argentina, Salta, *Cano & Alonso 8512* (MUB), (1680) **PX711863**, **PX898030**, **PX872816**, **PX704501**, **PZ069115**; Brazil (1), Mato Grosso, *Camara et al. 2819* (MUB), (994) **PX711864**, **PX897986**, **PX872812**, **PX704502**, **PZ069094**; Brazil (2), Mato Grosso, *Camara et al. 2821* (MUB), (1690) **PX517181**, **PX897987**, **PX872817**, **PX704503**, **PZ069114**. *Tuerckheimia svihlae* (E.B. Bartram) R.H. Zander, USA (1), Florida, *Cash & Rapp s.n.* (DUKE), -, -, -, HM147791, HM147817; USA (2), Tennessee, *Anderson 24791* (DUKE), -, -, -, -, AY796281; *Tuerckheimia valeriana* (E.B. Bartram) R.H. Zander, Costa Rica, *Bryotheca Goettingensis Fasc. 9, No. 38* (GOET), AY950350, -, AY950441, AY950396, AY854431. *Weissia condensa* (Voit) Lindb., Spain (1), Murcia, *Cano 6227* (MUB), KT380181, KT380255, KT380331, -, KT380406; Spain (2), Granada, *Cano 791* (MUB), -, -, -, -, AY796241. *Weissia controversa* Hedw., Portugal, Baixo Alentejo, *Cano 6259* (MUB), KT380183, KT380257, KT380333, -, KT380408; Switzerland, Ascona, *Callaghan DA DC240812* (Priv. Herb. D.A. Callaghan), -, -, MH545593, -, MH545638.
