# Supplementary material for: Hidden diversity in the Trichostomum brachydontium complex (Pottiaceae, Bryophyta) revealed by integrative taxonomy
Source: Front Plant Sci. 2026 Apr 21;17:1822444. doi: 10.3389/fpls.2026.1822444 (PMC13139172; doi:10.3389/fpls.2026.1822444)
Supplement: Supplementary file 1 [file SupplementaryFile1.zip › Supplementary_material/Supplementary_TABLE S2.docx]

**TABLE S2** Measured variables grouped by type with their identification codes; units provided when applicable.

| **Quantitative variables** | **ID** | **Qualitative variables** | **ID** |
| --- | --- | --- | --- |
| Plant length (mm) | 1 | Central strand development | 42 |
| Stem diameter (µm) | 2 | Hyalodermis development | 43 |
| Number of sclerodermis layers | 3 | Orientation of stem leaves when dry | 44 |
| Relative border extension (%) | 4 | Leaf shape | 45 |
| Leaf length (mm) | 5 | Papillae development on leaf margin | 46 |
| Leaf base width (mm) | 6 | Differentiation of teeth in transition zone | 47 |
| Leaf median width (mm) | 7 | Color of lamina in KOH reaction | 48 |
| Leaf apex width (mm) | 8 | Curvature of leaf margin in upper third | 49 |
| Costa base width (µm) | 9 | Leaf apex shape | 50 |
| Costa apex width (µm) | 10 | Apical lamina extension toward mucro | 51 |
| Mucro length (µm) | 11 | Orientation of mucro relative to leaf | 52 |
| Number of guide cells at base | 12 | Ventral costa surface cells shape | 53 |
| Number of guide cells at the middle | 13 | Costa shape at base (cross-section) | 54 |
| Number of guide cells at apex | 14 | Costa shape at the middle (cross-section) | 55 |
| Number of guide cells layers at base | 15 | Costa shape at upper (cross-section) | 56 |
| Number of ventral stereid layers at the middle | 16 | Differentiation of pairs of guide cells at the edge of the costa at base (cross-section) | 57 |
| Number of dorsal stereid layers at the middle | 17 | Lower basal cells shape | 58 |
| Length of ventral costa surface cells at middle (cross-section) (µm) | 18 | Marginal basal cells shape | 59 |
| Length of dorsal costa surface cells at middle (cross-section)  (µm) | 19 | Central basal cells shape | 60 |
| Basal hyaline zone (%) | 20 | Central basal cells wall thickness | 61 |
| Transition zone (%) | 21 | Juxtacostal basal cells shape | 62 |
| Length of lower basal cells (µm) | 22 | Juxtacostal basal cells wall thickness | 63 |
| Width of lower basal cells (µm) | 23 | Transition cells shape | 64 |

| Length of marginal basal cells (µm) | 24 | Meddian and upper laminal cells shape | 65 |
| --- | --- | --- | --- |
| Width of marginal basal cells (µm) | 25 | Papillae shape of the upper laminal cells | 66 |
| Length of central basal cells (µm) | 26 | Meddian and upper marginal laminal cells shape | 67 |
| Width of central basal cells (µm) | 27 |  |  |
| Juxtacostal basal cells length (µm) | 28 |  |  |
| Width of juxtacostal basal cells (µm) | 29 |  |  |
| Length of transition cells (µm) | 30 |  |  |
| Width of transition cells (µm) | 31 |  |  |
| Length of middle marginal laminal cells (µm) | 32 |  |  |
| Width of middle marginal laminal cells (µm) | 33 |  |  |
| Width of middle laminal cells (µm) | 34 |  |  |
| Numbers of papillae in middle laminal cells | 35 |  |  |
| Length of papillae in middle laminal cells (µm) | 36 |  |  |
| Length of upper marginal laminal cells (µm) | 37 |  |  |
| Width of upper marginal laminal cells (µm) | 38 |  |  |
| Width of upper laminal cells (µm) | 39 |  |  |
| Numbers of papillae in upper laminal cells | 40 |  |  |
| Length of papillae in upper laminal cells (µm) | 41 |  |  |
