# Supplementary material for: Hidden diversity in the Trichostomum brachydontium complex (Pottiaceae, Bryophyta) revealed by integrative taxonomy
Source: Front Plant Sci. 2026 Apr 21;17:1822444. doi: 10.3389/fpls.2026.1822444 (PMC13139172; doi:10.3389/fpls.2026.1822444)
Supplement: Supplementary file 1 [file SupplementaryFile1.zip › Supplementary_material/Supplementary_TABLE S3.docx]

**TABLE S3** List of qualitative variables with their identification codes (ID) and state levels.

| **ID** | **State (n)** | **1** | **2** | **3** | **4** | **5** | **6** | **7** | **8** |
| --- | --- | --- | --- | --- | --- | --- | --- | --- | --- |
| 42 | 3 | Not developed | Underdeveloped | Well-developed |  |  |  |  |  |
| 43 | 3 | Not developed | Complete | Incomplete |  |  |  |  |  |
| 44 | 4 | Imbricate and contorted | Incurved | Incurved and crispate | Twisted and crispate |  |  |  |  |
| 45 | 8 | Oblong-lingulate | Lanceolate | Oblong-lancolate | Elliptical-lanceolate | Lingulate | Linear | Linear-lanceolate | Elliptical |
| 46 | 3 | Entire | Papillose | Papillose-crenulate |  |  |  |  |  |
| 47 | 2 | Yes | No |  |  |  |  |  |  |
| 48 | 3 | Yellow | Yellow-orange | Orange |  |  |  |  |  |
| 49 | 3 | Incurved | Plane-inflexed | Inflexed |  |  |  |  |  |
| 50 | 4 | Acute | Obtuse | Acuminate | Rounded |  |  |  |  |
| 51 | 2 | Yes | No |  |  |  |  |  |  |
| 52 | 2 | Straight | Reflexed |  |  |  |  |  |  |
| 53 | 3 | Rectangular | Quadrate | Subquadrate |  |  |  |  |  |
| 54 | 3 | Biconvex | Plane-convex | Circular |  |  |  |  |  |
| 55 | 3 | Biconvex | Plane-convex | Circular |  |  |  |  |  |
| 56 | 3 | Biconvex | Plane-convex | Circular |  |  |  |  |  |
| 57 | 2 | Yes | No |  |  |  |  |  |  |
| 58 | 2 | Rectangular | Oblong |  |  |  |  |  |  |
| 59 | 2 | Rectangular | Quadrate |  |  |  |  |  |  |
| 60 | 4 | Oblong-hexagonal | Rectangular | Oblong | Linear |  |  |  |  |
| 61 | 2 | Thin-walled | Thick-walled |  |  |  |  |  |  |
| 62 | 3 | Linear | Rectangular | Quadrate |  |  |  |  |  |
| 63 | 2 | Thin-walled | Thick-walled |  |  |  |  |  |  |
| 64 | 5 | Quadrate | Rectangular | Rounded | Oblong | Linear |  |  |  |
| 65 | 5 | Subquadrate | Rectangular | Quadrate | Rounded | Oblate |  |  |  |

| 66 | 6 | Absent | Coroniform and  pedicellate | Coroniform not  pedicellate | Bifurcate | Simple | Simple and  bifurcate |
| --- | --- | --- | --- | --- | --- | --- | --- |
| 67 | 4 | Oblate | Rectangular | Quadrate | Subquadrate |  |  |
