# Supplementary material for: Hidden diversity in the Trichostomum brachydontium complex (Pottiaceae, Bryophyta) revealed by integrative taxonomy
Source: Front Plant Sci. 2026 Apr 21;17:1822444. doi: 10.3389/fpls.2026.1822444 (PMC13139172; doi:10.3389/fpls.2026.1822444)
Supplement: Supplementary file 1 [file SupplementaryFile1.zip › Supplementary_material/Supplementary_TABLE S4.docx]

**TABLE S4** Confusion matrix of the Linear Discriminant Analysis (LDA) model with 10-fold cross-validation. Rows represent actual groups from the phylogenetic analysis (A-K), columns represent predicted groups (1-11). Diagonal values indicate correct classifications; off-diagonal values (in red) indicate misclassifications.

|  |  |  | | | | | | | | | | |
| --- | --- | --- | --- | --- | --- | --- | --- | --- | --- | --- | --- | --- |
|  |  | **A** | **B** | **C** | **D** | **E** | **F** | **G** | **H** | **I** | **J** | **K** |
|  | **A** | **4** | 0 | 0 | 0 | **1** | 0 | 0 | **1** | **1** | 0 | **1** |
|  | **B** | 0 | **8** | 0 | 0 | 0 | 0 | 0 | 0 | 0 | 0 | 0 |
|  | **C** | 0 | 0 | **14** | 0 | 0 | 0 | 0 | 0 | 0 | 0 | 0 |
|  | **D** | 0 | 0 | 0 | **4** | 0 | 0 | 0 | 0 | 0 | 0 | 0 |
|  | **E** | 0 | 0 | 0 | 0 | **16** | 0 | 0 | 0 | 0 | 0 | 0 |
|  | **F** | 0 | 0 | 0 | 0 | 0 | **18** | 0 | 0 | 0 | 0 | 0 |
|  | **G** | 0 | 0 | 0 | 0 | 0 | 0 | **6** | 0 | 0 | 0 | 0 |
|  | **H** | 0 | 0 | 0 | 0 | 0 | 0 | 0 | **54** | 0 | 0 | 0 |
|  | **I** | 0 | 0 | 0 | 0 | 0 | 0 | 0 | 0 | **12** | 0 | 0 |
|  | **J** | 0 | 0 | 0 | 0 | 0 | 0 | 0 | 0 | 0 | **6** | 0 |
|  | **K** | 0 | 0 | 0 | 0 | 0 | 0 | 0 | 0 | 0 | 0 | **45** |
